# Allometric fat mass index and alanine aminotransferase attenuate the associations of platelet parameters with lung cancer risk

**DOI:** 10.1038/s41598-024-78281-x

**Published:** 2024-11-01

**Authors:** Sofia Christakoudi, Konstantinos K. Tsilidis, Marc J. Gunter, Elio Riboli

**Affiliations:** 1 Department of Epidemiology and Biostatistics, School of Public Health, Imperial College London, White City Campus, 90 Wood Lane, London, W12 0BZ UK; 2 Department of Hygiene and Epidemiology, University of Ioannina School of Medicine, Ioannina, Greece

**Keywords:** Platelet count, MPV, Body composition, Fat mass, ALT, Liver function tests, Interaction, Lung cancer risk, Lung cancer, Obesity, Liver, Risk factors

## Abstract

We have previously shown that body mass index attenuates a positive association of platelet count (PLT) and inverse of mean platelet volume (MPV) with lung cancer risk in men. It is unclear whether fat mass, lean mass, or liver function tests (LFTs) show similar attenuations. Using bioelectrical impedance measurements (UK Biobank cohort) and multivariable Cox proportional hazards models, we examined the associations of allometric fat-mass index (AFI, fat mass adjusted for height), allometric lean-mass index (ALI, fat-free mass adjusted for height and fat mass), and LFTs with lung cancer risk and their multiplicative and additive interactions with platelet parameters. Based on 1573 lung cancer cases in men and 1473 in women with body composition measurements (1541 in men; 1428 in women with biomarker measurements), AFI in women, ALI in both sexes, alanine aminotransferase (ALT) and total bilirubin in men were inversely associated, while gamma-glutamyl transferase in men and alkaline phosphatase in both sexes were positively associated with lung cancer risk. Only AFI and ALT interacted inversely with PLT and positively with MPV in men. The attenuation of the associations of platelet parameters with lung cancer risk by high-AFI and high-ALT in men suggests that adiposity-related factors hinder lung-cancer-related platelet associations.

**Supplementary Information:**

The online version contains supplementary material available at 10.1038/s41598-024-78281-x.

## Introduction

The lung is a reservoir of circulating megakaryocytes and a major site of platelet production^1^. Platelets secrete microparticles and exosomes containing growth and angiogenic factors and thus are involved in regulation of local and distant interactions between the tumour and the host, epithelial to mesenchymal transition, tumour growth, and metastasis^2^. Correspondingly, higher platelet count (PLT) is associated with higher lung cancer risk for at least a decade prior to lung cancer diagnosis in both sexes^3,4^. Paradoxically, however, although obesity is associated with higher risk of venous thrombosis^5^, body mass index (BMI) is positively associated with PLT only in women and is inversely associated with PLT in men − more strongly inversely among individuals with higher alcohol consumption and smokers^6^. BMI is further inversely associated with lung cancer risk for both sexes in ever smokers and not in never smokers^7,8^, which may be entirely due to residual confounding from smoking but could potentially be determined by a mechanism gaining relevance only in smokers. More importantly, BMI appears to attenuate the positive association of PLT with lung cancer risk specifically in men^3^. We have previously proposed that the inverse interaction of obesity with PLT may be explained by a contribution of obesity to liver fibrosis and a destruction of circulating platelets following the development of non-alcoholic fatty liver disease (NAFLD)^3^. This process could be prominent only in men because female sex and oestrogens are apparently protective against NAFLD-related fibrosis^9^.

Low PLT, however, is a feature of any type of liver fibrosis, not specifically one related to obesity^10^, while alanine-aminotransferase (ALT) is characteristically high in NAFLD and gamma-glutamyl transferase (GGT) is particularly high in relation to alcohol consumption and alcohol-related liver damage^11^. A question, therefore, emerges whether liver function tests could attenuate the association of PLT with lung cancer risk in the same way that BMI does, and whether they all do this in a similar way, or whether such an attenuation is specific to ALT. Furthermore, if the attenuation is specifically related to liver fat infiltration, as opposed to any other liver damage, a similar attenuation may be noted only for fat mass and not for lean mass.

Mature platelets are smaller than platelet precursors^12^ and, correspondingly, PLT is inversely associated with mean platelet volume (MPV), which in turn is inversely associated with lung cancer risk and this association is attenuated in obesity^3^. Associations of PLT with cancer risk, however, are not always accompanied with associations of MPV with cancer risk in the opposite direction, as PLT is positively associated with prostate cancer risk but there is no evidence for an inverse association with MPV^13^. A further question, therefore, arises whether fat mass and ALT would show positive interactions with MPV (in a complementary pattern to the inverse interactions with PLT) in the same way that BMI does.

To answer the questions highlighted above, we investigated the prospective associations of body composition measured with bioelectrical impedance analysis (BIA) and liver function tests with lung cancer risk and their interactions with PLT and MPV in men and women from the UK Biobank cohort. To account for the positive correlations of body composition measurements with height and with each other, we developed allometric body composition indices.

We hypothesised that only fat mass and ALT in men will attenuate the associations of PLT and MPV with lung cancer risk and no such attenuations will be observed in women, or for fat-free mass, or for other liver function tests.

## Methods

### Study population

UK Biobank participants (some half a million in total from England, Scotland, and Wales) were recruited between the years 2006 and 2010, when aged 40 to 70 years^14^. In this study, we have used a similar subset as in our previous study^3^, restricting to participants with self-reported white ancestry, and excluding pregnant women and participants with a mismatch between the genetic and self-reported sex, missing or extreme anthropometric measurements, prevalent cancer at recruitment, using antihemorrhagic agents, with missing platelet measurements, or with missing all BIA and liver function measurements − total excluded 36,833 (16.1%) men and 54,633 (20.0%) women (Supplementary Table S1).

### Lung cancer ascertainment

Cancer ascertainment was based on linkage of UK Biobank to the national cancer registries of the United Kingdom. Lung cancer cases were defined as first primary cancer after recruitment with code C34 from the 10th version of the International Statistical Classification of Diseases (ICD10) and malignant behaviour (behavioural code 3 or 5)^15^. We censored follow-up at the date of diagnosis for first primary incident cancers in locations other than the lung (including skin squamous-cell carcinomas but excluding skin basocellular carcinomas), as well as for first primary incident lung cancers with rare morphology (codes 8710, 8800, 8801, 8990, 9050, 9120, 9133, 9591, 9680, 9699). We censored follow-up for participants remaining cancer-free at the earlier of the date of death or the last complete cancer registry (31st December 2016 for Wales; 31st March 2020 for England and Scotland).

### Anthropometric measurements and derived indices

Weight and height, measured according to pre-defined protocols^16^, and BIA of total fat mass (FM, kg) and total fat-free mass (FFM, kg), measured with Tanita BC-418MA Body Fat Analyser (Tanita Corp, Tokyo, Japan), were obtained by dedicated UK Biobank technicians at recruitment.

BMI was calculated as weight (kg) divided by height squared (m), which adjusts weight for height. In analogy to BMI, we derived allometric body composition indices to adjust for the positive correlations with height, more substantial for FFM than for FM (Supplementary Figure S1). In addition, we incorporated in the allometric index for FFM an adjustment for FM, to account for the substantial positive correlation between them (Supplementary Figure S1) and the overestimation of FFM by BIA in obesity^17,18^. To derive the scaling power coefficients (separately for men and women), we regressed log-transformed FM on log-transformed height (for the allometric fat-mass index, AFI) and regressed log-transformed FFM on log-transformed height and log-transformed FM (for the allometric lean-mass index, ALI) (Supplementary Table S2). We calculated AFI and ALI as follows:

$$\:{AFI}_{men}=FM\:\left(kg\right)*Height\:\left(m\right){\:}^{-1.0290}$$

$$\:{AFI}_{women}=FM\:\left(kg\right)*Height\:\left(m\right){\:}^{-1.3582}$$

$$\:{ALI}_{men}=FFM\:\left(kg\right)*Height\:\left(m\right){\:}^{-1.8122}*\:{FM\:\left(kg\right)\:}^{-0.1481}$$

$$\:{ALI}_{women}=FFM\:\left(kg\right)*Height\:\left(m\right){\:}^{-1.1960}*\:{FM\:\left(kg\right)\:}^{-0.1670}$$

### Platelet parameters and liver function tests

Blood samples were obtained throughout the day at recruitment, irrespective of fasting status. Platelet parameters were measured within 24 h of blood draw on Beckman Coulter LH750 analysers^19^. Liver function tests were measured in serum on Beckman Coulter AU5800 analysers^20^. We examined liver enzymes measured with enzymatic rate assays: ALT, aspartate aminotransferase (AST), GGT, and alkaline phosphatase (ALP), as well as direct (conjugated) bilirubin (BLD) and total bilirubin (BLT), measured with colorimetric assays. Values outside the limits of detection were few and were imputed with half the lowest detected level or the highest detected level, except for BLD, which was below the limit of detection for 13.5% of participants and we imputed this with quantile regression imputation of truncated left-censored data (QRILC)^21^ (Supplementary Table S3).

We log-transformed all platelet parameters and liver function tests, to mitigate right-skewness of their distributions.

### Statistical analysis

We used STATA-13 for the statistical analyses and R version 4.1.3^22^ for data management. For comparability, we transformed all anthropometric indices, platelet parameters, and liver function tests to sex-specific z-scores (value minus mean divided by standard deviation, SD). We calculated pairwise partial Pearson correlation coefficients (r) adjusted for age at recruitment. We performed all analyses separately in men and women, as the interactions with platelet parameters were previously observed only in men^3^. We have presented associations with ALT and GGT in the main analyses (as indicators of NAFLD and alcohol-related fatty-liver disease, correspondingly) and associations with the remaining liver function tests in the Supplementary Material.

First, we examined as exposures anthropometric indices and liver function tests, each individually on a continuous scale, and interpreted hazard ratios (HR) per one SD increase. To evaluate sex differences in HR estimates (p_sex_), we used the augmentation method of Lunn and McNeil^23^ and compared with a likelihood ratio test a full interaction model with a model omitting the interaction only for the examined variable of interest. This approach takes into account that in separate analyses of men and women the interaction with sex applies not only to the examined variable of interest but also to all covariates. For the full interaction model, we created a single dataset (including men and women) with two sets of variables – the first set including for all variables their values for men and zeros for women and the second set including for all variables their values for women and zeros for men. Examining the full interaction dataset with a Cox proportional hazards model and sex as a stratifying factor recreated the HR estimates of the separate analyses for men and women. For the model omitting the interaction, a single copy of the examined variable of interest (one at a time) was included with values for both men and women (no zeros). We then examined separately for each anthropometric index or liver function test multiplicative interactions with each of PLT or MPV individually on a continuous scale. For exposures with evidence for a multiplicative interaction, we examined in separate models associations of PLT or MPV with lung cancer risk in groups defined by the sex-specific tertiles of the relevant anthropometric index or liver function test and tested heterogeneity with the augmentation method of Lunn and McNeil^23^, comparing the highest sex-specific tertile group with the combined group of the middle and lowest tertiles (p_heterogeneity_). To examine additive interactions, we used two-way cross-classifications between either PLT or MPV (dichotomised at the sex-specific median) and one at a time anthropometric index or liver function test (dichotomised at the upper sex-specific tertile cut-off). We calculated the relative excess risk from interaction (RERI)^24^ and obtained confidence intervals and p-values with the delta method applied in function **nlcom** in STATA-13^25^:

RERI = HR_High−High_ – HR_High−Low_ – HR_Low−High_ + 1.

To explore sex differences, we used three-way cross-classifications, grouping additionally by sex.

For all associations and interactions, we derived HRs and 95% confidence intervals (CI) with delayed-entry Cox proportional hazards models, which are conditional on surviving cancer-free to recruitment and thus account for left-truncation. We used age as the underlying time scale: origin − date of birth; entry time − date at recruitment; exit time − the earliest of the date of diagnosis of the first primary incident cancer, or death, or last complete follow-up. As in our previous study, the selection of non-smoking covariates was based on reports for associations with platelet parameters and lung cancer risk^3^. In this study, we examined pairwise associations of candidate covariates with AFI, ALI, PLT, ALT, GGT, and lung cancer risk, separately in men and women, and retained those covariates associated with at least one of the exposures and the outcome (Supplementary Figure S2, see also details of the definition of covariates in the legend).

All main models were stratified by age at recruitment (five-year categories), region of the assessment centre, and smoking status and intensity (never smoked; just tried; former occasional; former regular quit ≥ 20 years; former regular quit ≥ 10 years; former regular quit < 10 years; current occasional; current regular ≤ 10 cigarettes/day; current regular > 10 cigarettes/day). All models were adjusted for height (sex-specific z-scores), weight gain within the year preceding recruitment (no/yes), alcohol consumption (≤ 3 times/month; ≤4 times/week; daily), physical activity (less active; moderately active; very active), Townsend deprivation index quintiles (as proxy of socio-economic status), family history of lung cancer (no/yes), time of blood collection (< 12:00; 12:00 to < 16:00; ≥16:00), fasting time (0–2 h; 3–4 h; ≥5 h), self-reported diabetes, use of lipid lowering drugs, antihypertensive drugs, antiaggregant/anticoagulants, and paracetamol (no/yes) and, for women, a combined variable reflecting menopausal status and hormone replacement therapy (HRT) use (pre-menopausal; post/unknown menopause never HRT; post/unknown menopause past HRT; post/unknown menopause current HRT). All variables were measured at baseline and were not updated during the follow-up period. We tested the proportional hazards assumption based on Schoenfeld residuals.

We replaced the limited number of missing values for covariates (< 2%) with the median category for each sex. Tests of statistical significance were two-sided and were evaluated at nominal statistical significance (*p* < 0.05), to compensate for the higher power requirements of tests for interaction.

### Sensitivity analyses

In sensitivity analyses, we compared allometric and traditional body composition indices with and without adjustment for height and with mutual adjustment (combining in the same model AFI with ALI or FM with FFM). We additionally adjusted for PLT or MPV the main models examining associations with lung cancer risk. To explore the influence of the adjustment for smoking and covariates, we compared the main models examining associations and interactions with models stratified only by age or stratified by age and smoking status and intensity, omitting the adjustment for covariates. We further examined associations and interactions in groups according to smoking status: non-smokers (combining never and former smokers due to the limited number of cases in never smokers) and current smokers. Last, to examine the potential influence of reverse causality, we excluded participants with less than eight years of follow-up and correspondingly lagged the entry time with eight years.

## Results

### Cohort characteristics

All body composition measurements were available in a dataset including 188,946 men (1573 lung cancer cases) and 216,101 women (1473 cases) (Table 1). All liver function tests were available in a largely overlapping dataset (> 95%) including 183,104 men (1541 cases) and 208,339 women (1428 cases) (Supplementary Table S4). The median time to diagnosis was ≈ 6.5 years and over 75% of lung cancer cases were diagnosed more than 3 years post recruitment. In groups according to smoking status, BMI and AFI were highest in former smokers (with little differences between never and current smokers), while ALT was lowest, but GGT and PLT were highest in current smokers, with a similar pattern in men and women (Table 1). FM was correlated weakly positively with height (*r* ≈ 0.15), but FFM was correlated substantially positively with height and with FM (*r* = 0.49 to 0.69), while the correlations between AFI, ALI, and height were minimal (*r* = − 0.05 to 0.10) (Supplementary Figure S1). BMI and AFI were correlated similarly positively with both ALT and GGT (*r* ≈ 0.30), which were also correlated substantially positively with each other (*r* ≈ 0.55).

**Table 1:** Anthropometric characteristics, platelet parameters, and liver function tests of study participants.

	Total	Never smokers	Former smokers	Current smokers	*p*_smoking_
Men					
Cohort: n (%)	188,946	91,987 (48.7)	74,041 (39.2)	22,918 (12.1)	
Cases: n (rate)	1573 (810)	138 (144)	761 (1014)	674 (2916)	
Time to diagnosis^a^	6.4 (3.4–8.9)	6.6 (3.3–8.8)	6.4 (3.4–9.0)	6.3 (3.5–8.8)	0.961
Age (years)^b^	57.2 (8.1)	56.0 (8.2)	59.3 (7.6)	55.5 (8.2)	3*10^− 188^
Height (cm)^b^	175.9 (6.8)	176.2 (6.8)	175.7 (6.7)	175.5 (6.8)	1*10^− 67^
BMI (kg/m^2^)^b^	27.8 (4.0)	27.5 (3.9)	28.4 (4.0)	27.4 (4.2)	2*10^− 83^
Total Fat Mass (kg)^**b**^	22.2 (7.9)	21.4 (7.7)	23.5 (7.9)	21.4 (8.1)	3*10^− 125^
Total Fat-Free Mass (kg)^b^	63.9 (7.6)	63.8 (7.5)	64.1 (7.5)	63.1 (8.0)	6*10^− 13^
AFI^b^	12.4 (4.4)	12.0 (4.3)	13.2 (4.4)	12.0 (4.5)	5*10^− 146^
ALI^b^	14.60 (1.07)	14.63 (1.07)	14.56 (1.06)	14.56 (1.14)	3*10^− 38^
PLT (*10^9^/L)^c^	232 (146–370)	231 (146–365)	232 (145–370)	239 (147–390)	4*10^− 74^
MPV (fL)^c^	9.22 (7.40–11.49)	9.22 (7.41–11.46)	9.23 (7.39–11.52)	9.25 (7.41–11.54)	3*10^− 5^
ALT (IU/L)^c^	24.8 (10.5–58.4)	24.7 (10.6–57.6)	25.3 (10.8–59.3)	23.5 (9.5–58.2)	2*10^− 10^
GGT (IU/L)^c^	36.2 (11.1–118.5)	33.9 (10.7–107.4)	38.2 (11.6–125.4)	39.5 (11.3–137.8)	< 1*10^− 311^
Women					
Cohort: n (%)	216,101	127,775 (59.1)	69,379 (32.1)	18,947 (8.8)	
Cases: n (rate)	1473 (649)	272 (202)	634 (875)	567 (2888)	
Time to diagnosis^a^	6.6 (4.1–9.0)	6.4 (3.9–9.2)	6.5 (4.0–8.9)	6.8 (4.3–9.1)	0.586
Age (years)^b^	56.9 (8.0)	56.6 (8.0)	57.9 (7.7)	54.8 (8.0)	3*10^− 4^
Height (cm)^b^	162.6 (6.2)	162.6 (6.2)	162.8 (6.2)	162.5 (6.3)	0.014
BMI (kg/m^2^)^b^	26.9 (4.8)	26.8 (4.8)	27.2 (4.8)	26.7 (4.7)	6*10^− 19^
Total Fat Mass (kg)^b^	26.7 (9.4)	26.4 (9.3)	27.4 (9.5)	26.1 (9.5)	4*10^− 22^
Total Fat-Free Mass (kg)^b^	44.5 (4.8)	44.4 (4.7)	44.7 (4.8)	44.4 (4.9)	4*10^− 19^
AFI^b^	13.8 (4.8)	13.6 (4.8)	14.1 (4.8)	13.5 (4.8)	2*10^− 19^
ALI^b^	14.49 (1.00)	14.48 (1.00)	14.48 (1.00)	14.54 (1.01)	1*10^− 4^
PLT (*10^9^/L)^c^	260 (165–409)	259 (165–407)	260 (165–408)	264 (164–424)	4*10^− 19^
MPV (fL)^c^	9.31 (7.46–11.62)	9.30 (7.46–11.60)	9.30 (7.46–11.61)	9.38 (7.49–11.76)	2*10^− 14^
ALT (IU/L)^c^	18.2 (7.9–41.8)	18.1 (7.9–41.4)	18.7 (8.1–43.1)	17.2 (7.5–39.7)	0.001
GGT (IU/L)^c^	23.8 (7.6–74.4)	23.2 (7.5–71.4)	24.6 (7.8–77.9)	25.5 (8.0–80.9)	7*10^− 161^

AFI: allometric fat-mass index; ALI: allometric lean-mass index; ALT: alanine aminotransferase; BMI: body mass index; GGT: gamma-glutamyl transferase; MPV: mean platelet volume; PLT: platelet count; n (%): number of participants per group (percentage from total per sex); n (rate): number of lung cancer cases per group (incidence rate per 1*10^6^ person years).

^a^ median (interquartile range: 25th -75th centile); ^b^ mean (standard deviation); ^c^ geometric mean (95% reference range).

Anthropometric characteristics and platelet parameters are summarised for the dataset with available all body composition measurements. ALT and GGT are summarised for the dataset with available all liver function tests (additional summaries for this dataset are shown in Supplementary Table S4).

Smoking status groups per sex were compared with analysis of variance (after log-transformation for biomarkers) (p_smoking_).

### Associations of anthropometric indices and liver function tests with lung cancer risk

**Fig. 1:**
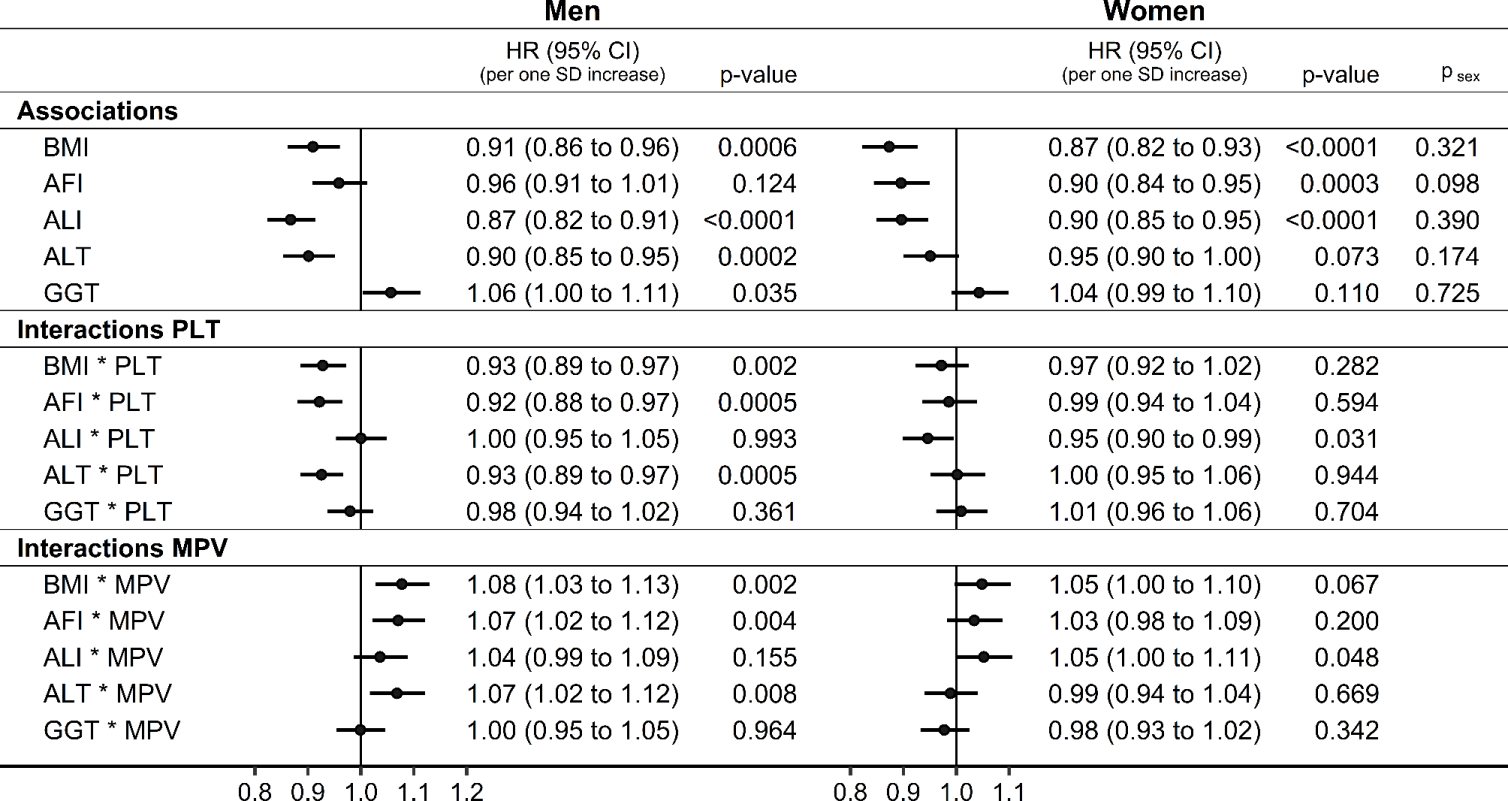
Associations of anthropometric indices and liver function tests with lung cancer risk and multiplicative interactions with platelet parameters. AFI: allometric fat-mass index; ALI: allometric lean-mass index; ALT: alanine aminotransferase; BMI: body mass index; CI: confidence interval; GGT: gamma-glutamyl transferase; HR: hazard ratio; MPV: mean platelet volume; PLT: platelet count; SD: standard deviation; cases: number of lung cancer cases; rate: incidence rate per 1*10^6^ person years; p-value: Wald test for the individual term (associations) or for the individual multiplicative interaction term (interactions); p_sex_: p-value comparing the association with lung cancer risk between men and women with the augmentation method of Lunn and McNeil^23^. Cox proportional hazards models with exposure each anthropometric index or liver function test individually (associations), or additionally including a multiplicative interaction term between the examined exposure of interest and either PLT or MPV (sex-specific z-scores, value minus mean divided by SD, after log-transformation for biomarkers), stratified by age at recruitment, region, and smoking status and intensity, and adjusted for height, recent weight gain, alcohol consumption, physical activity, Townsend deprivation index, family history of lung cancer, time of blood collection, fasting time, diabetes, and use of lipid-lowering drugs, antihypertensive drugs, antiaggregant/anticoagulants, and paracetamol, and in women, menopausal status and hormone replacement therapy use. Associations and interactions with other liver function tests are shown in Supplementary Figure S4.

AFI showed little evidence for association with lung cancer risk in men (HR = 0.96; 95%CI: 0.91–1.01 per one SD increase) but was inversely associated in women (HR = 0.90; 95%CI: 0.84–0.95), while ALI was inversely associated in both men (HR = 0.87; 95%CI: 0.82–0.91) and women (HR = 0.90; 95%CI: 0.85–0.95) (Fig. 1). Mutual adjustment of AFI, ALI, and height did not influence materially their associations with lung cancer risk, but mutual adjustment of FM, FFM, and height markedly shifter their associations with lung cancer risk − towards the positive for FM and height and towards the inverse for FFM (Supplementary Figure S3).

ALT was inversely associated (HR = 0.90; 95%CI: 0.85–0.95 per one SD increase) while GGT was positively associated (HR = 1.06; 95%CI: 1.00–1.11) with lung cancer risk in men, with similar associations but without nominal statistical significance in women (Fig. 1). There was little evidence for associations of AST or BLD with lung cancer risk, but ALP was positively associated in both sexes (stronger in men: HR = 1.19; 95%CI: 1.13–1.24; weaker in women: HR = 1.07; 95%CI: 1.01–1.13; p_sex_=0.007), while BLT was inversely associated with lung cancer risk only in men (HR = 0.90; 95%CI: 0.85–0.95) (Supplementary Figure S4).

In sensitivity analyses, further adjustment for PLT or MPV made no material difference to the described associations (Supplementary Figure S5). Adjustment for smoking and covariates was required to reveal the inverse associations with AFI but influenced little the inverse associations with ALI, while adjustment for smoking alone attenuated all associations with liver function tests, and most associations were stronger in current smokers but were largely directionally consistent and only partly attenuated for longer follow-up time (≥ 8 years) (Supplementary Figure S6).

### Interactions of anthropometric indices and liver function tests with platelet parameters

**Fig. 2:**
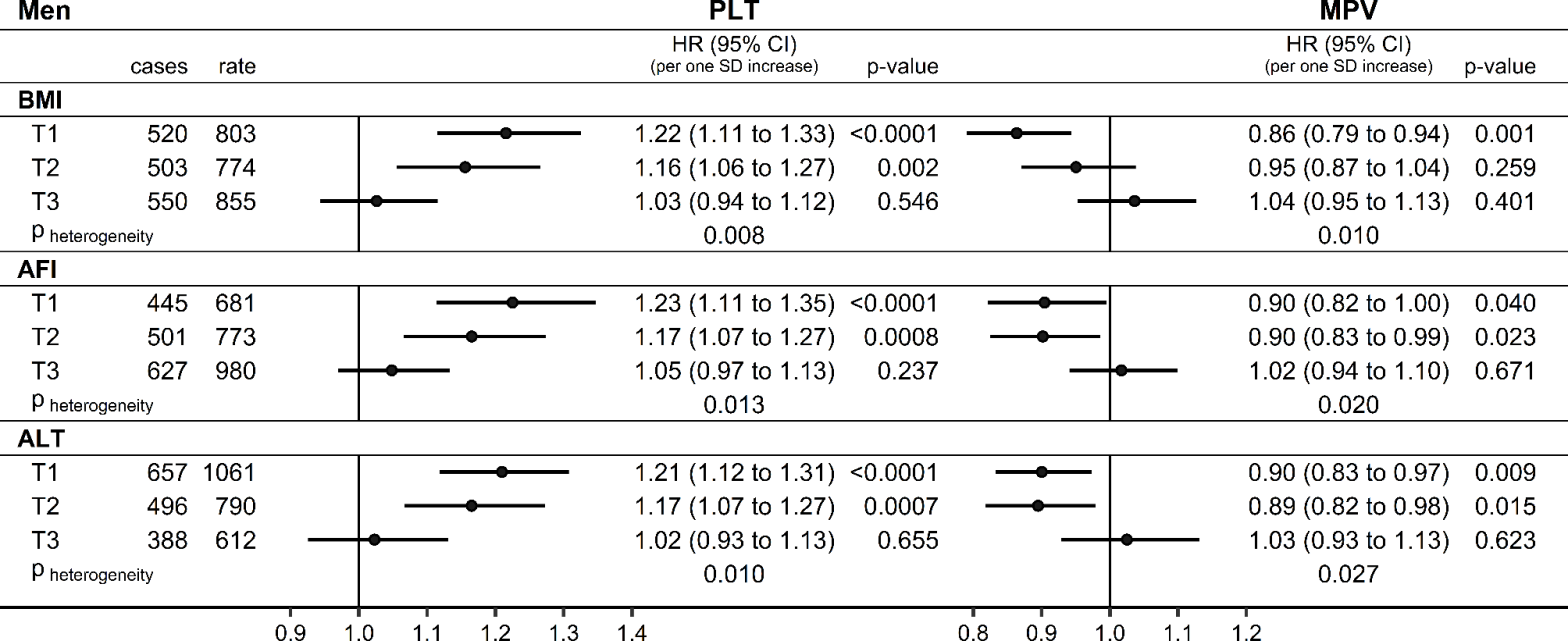
Associations of platelet parameters with lung cancer risk (men): groups by BMI, AFI, ALT tertiles. AFI: allometric fat-mass index (cut-offs: 10.293; 13.703); ALT: alanine aminotransferase (cut-offs: 20.25; 28.65 IU/L); BMI: body mass index (cut-offs: 25.806; 28.982 kg/m^2^); CI: confidence interval; HR: hazard ratio; MPV: mean platelet volume; PLT: platelet count; SD: standard deviation; T1-T3: sex-specific tertiles; cases: number of lung cancer cases; rate: incidence rate per 1*10^6^ person years; p-value: p-value from Wald test for the individual term; p_heterogeneity_: p-value comparing the association with lung cancer risk between the highest sex-specific tertile group and the combined group of the middle and lowest tertile with the augmentation method of Lunn and McNeil^23^. Cox proportional hazards models in groups according to tertiles of either BMI, AFI, or ALT in men, including as exposure either PLT or MPV (sex-specific z-scores, value minus mean divided by standard deviation after log-transformation), stratified by age at recruitment, region, and smoking status and intensity, and adjusted for height, recent weight gain, alcohol consumption, physical activity, Townsend deprivation index, family history of lung cancer, time of blood collection, fasting time, diabetes, and use of lipid-lowering drugs, antihypertensive drugs, antiaggregant/anticoagulants, and paracetamol.

As hypothesised, only AFI and ALT in men (in addition to BMI) showed substantial multiplicative interactions on a continuous scale with platelet parameters (Fig. 1) − inverse with PLT (HR = 0.92; 95%CI = 0.88 − 0.97 per one SD increase for AFI; HR = 0.93; 95%CI = 0.89 − 0.97 for ALT) and positive with MPV (HR = 1.07; 95%CI = 1.02 − 1.12 for AFI; HR = 1.07; 95%CI = 1.02 − 1.12 for ALT) (Fig. 1). Correspondingly, PLT was positively associated and MPV was inversely associated with lung cancer risk only for the lowest and middle tertiles but not for the highest tertile of BMI, AFI, or ALT in men (p_heterogeneity_<0.05 for all) (Fig. 2).

**Fig. 3:**
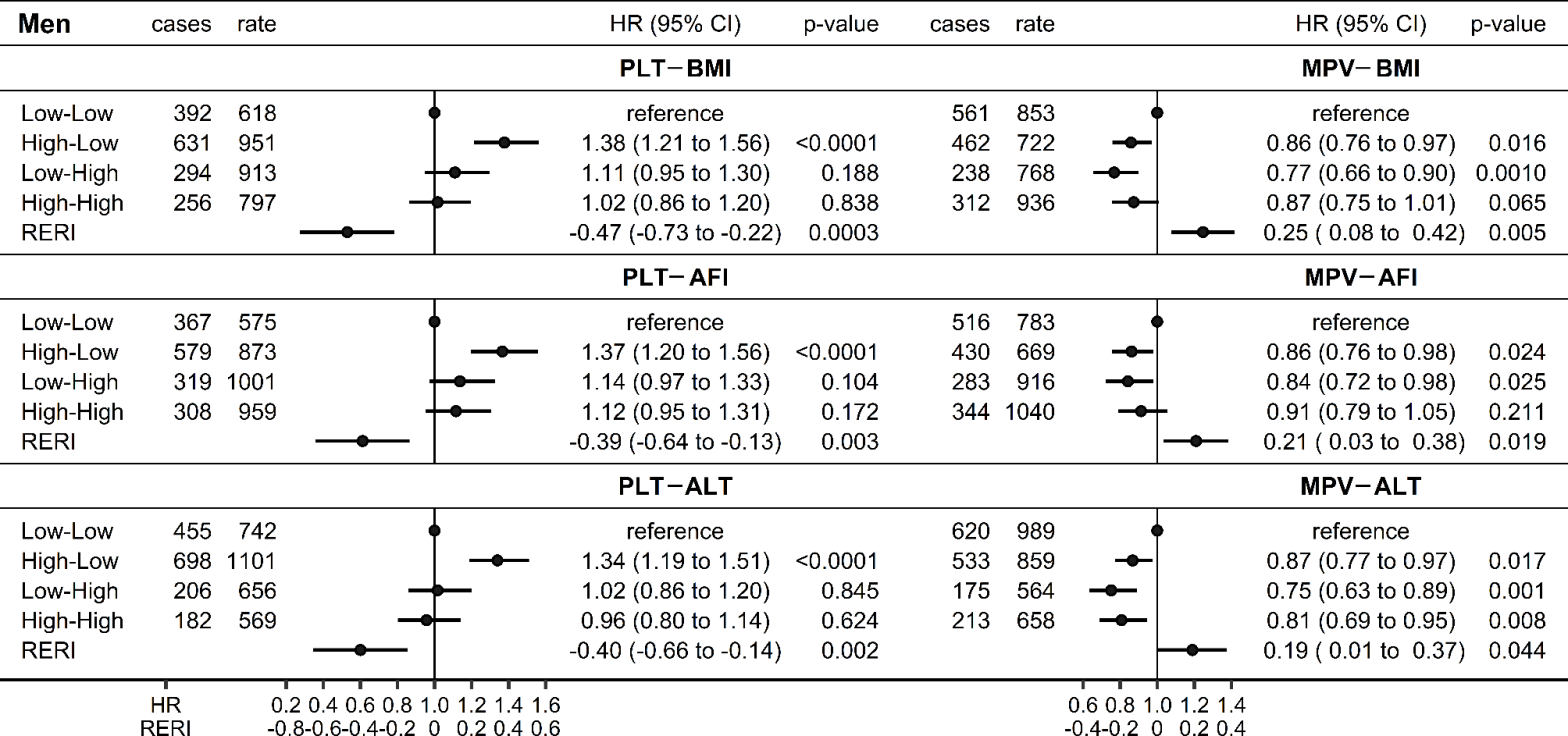
Additive interactions of body composition indices or alanine aminotransferase with platelet parameters (men). AFI: allometric fat-mass index (cut-off: ≥13.703); ALT: alanine aminotransferase (cut-off: ≥28.65 IU/L); BMI: body mass index (cut-off: ≥28.982 kg/m^2^); CI: confidence interval; HR: hazard ratio; MPV: mean platelet volume (cut-off: ≥9.17 fL); PLT: platelet count (cut-off: ≥234.0*10^9^/L); RERI: relative excess risk from interaction (additive interaction); cases: number of lung cancer cases; rate: incidence rate per 1*10^6^ person years; p-value: p-value for RERI derived with the delta method or p-value from Wald test for the individual term. Cox proportional hazards models including a cross-classification between either PLT or MPV (dichotomised at the sex-specific median) and one of BMI, AFI, or ALT in men (dichotomised at the upper sex-specific tertile cut-off), stratified by age at recruitment, region, and smoking status and intensity, and adjusted for height, recent weight gain, alcohol consumption, physical activity, Townsend deprivation index, family history of lung cancer, time of blood collection, fasting time, diabetes, and use of lipid-lowering drugs, antihypertensive drugs, antiaggregant/anticoagulants, and paracetamol.

In two-way cross-classifications in men, both AFI and ALT showed similar pattern to BMI. Thus, lung cancer risk was higher for high-PLT only for low-AFI or low-ALT (high-low group compared to the reference low-low group) and there were inverse additive interactions with PLT (RERI = − 0.39; 95%CI: −0.64 to − 0.13 for AFI; RERI = − 0.40; 95%CI: −0.66 to − 0.14 for ALT) (Fig. 3). A complementary pattern in the opposite direction was observed for MPV, with lower lung cancer risk for high-MPV compared to low-MPV only for low-AFI or low-ALT, and with positive additive interactions with MPV (RERI = 0.21; 95%CI: 0.03 to 0.38 for AFI; RERI = 0.19; 95%CI: 0.01 to 0.37 for ALT) (Fig. 3). PLT and MPV levels, however, did not differ according to the levels of BMI, AFI, or ALT (Supplementary Table S5). In women, PLT was positively associated with lung cancer risk irrespective of BMI, AFI, or ALT levels and there was no evidence for associations of MPV with lung cancer risk (Supplementary Figure S7).

In sensitivity analyses in men, the interactions with PLT and MPV were attenuated little by adjustment for smoking and covariates or in groups by smoking status and only the interactions of MPV with ALT were lost in non-smokers, the interactions of MPV with AFI were lost in current smokers, and the interactions of PLT with ALT were lost for longer follow-up time (≥ 8 years) (Supplementary Figure S8).

## Discussion

In this study, ALI accounted for the inverse association of BMI with lung cancer risk in men while both AFI and ALI showed inverse associations in women, but only AFI showed consistent multiplicative and additive interactions in men, inverse for PLT and positive for MPV. Among liver function tests, only ALT was inversely associated with lung cancer risk in men and showed multiplicative and additive interactions with platelet parameters. Although BLT was also inversely associated with lung cancer risk in men, there was no evidence for interactions, while GGT and ALP were positively associated with lung cancer risk in men and ALP also in women, without evidence for interactions with platelet parameters.

Our findings suggest that the attenuation by BMI of the associations of platelet parameters with lung cancer risk in men is unlikely to be explained by the same mechanism as the inverse association of BMI with lung cancer risk in both sexes, since an attenuation in men was observed only for AFI, while an inverse association in both sexes was observed only for ALI. Furthermore, the interactions of AFI and ALT and not ALI or other liver function tests with platelet parameters support the involvement of a factor related to fat accumulation rather than liver fibrosis, which would not be confined to NAFLD. We have shown in this study that even when platelet count was high, it was not associated with higher lung cancer risk when BMI, AFI, or ALT was also high. This makes it less likely that platelet-related lung cancer risk is lower in obesity due to lower platelet count, or due to lower liver production of coagulation factors, as the latter mechanism would not be specific for NAFLD either. Our findings instead suggest that a factor related to adipose tissue expansion may hinder the pro-oncogenic platelet associations. Such a factor is more likely to originate from peripheral as opposed to local adipose tissue, as adipose tissue accumulates only in the outer wall of large airways (“fatty airways”), contributing to local inflammatory processes, while there is no evidence for fat accumulation in the lung parenchyma^26^. If local adipose tissue is relevant, the interactions with platelet parameters may differ between adenocarcinomas and squamous cell carcinomas, which would be important to investigate with a larger sample size. The consistent positive interactions of AFI and ALI with MPV, opposite to the inverse interactions with PLT, are compatible with smaller platelet size reflecting platelet maturity^12^ and indicate that the interactions with PLT and MPV are likely related to functional and mature platelets. Although reverse causality cannot be ruled out, the positive association of PLT with lung cancer risk has been shown to be markedly stronger only within the last year prior to diagnosis, remaining reasonably stable between ten years and up to twelve months prior to diagnosis^4^. We have shown in this study that the interactions with platelet parameters were largely retained for over eight years prior to diagnosis.

An obesity-related mechanism hindering positive associations with platelets is apparently paradoxical because obesity is a proinflammatory and prothrombotic state^5^ and many platelet-related factors with potentially pro-oncogenic properties are higher in obesity^27,28^. There is, however, a mechanism that could potentially explain this paradox and this involves CD40 receptor and the related CD40 ligand (CD40L), which both belong to the tumour necrosis factor superfamily^29^. CD40L is either membrane bound or soluble (sCD40L) and originates mainly from blood platelets, where it is stored in granules and is released after platelet activation and degranulation^30^. In analogy to tumour associated macrophages and neutrophils, which have anti-neoplastic as well as pro-oncogenic phenotypes^31^, tumour associated platelets could either stimulate tumour growth and metastasis or suppress tumour progression by activating anti-neoplastic immune responses or by induction of cancer cell apoptosis^29^. More interestingly, while macrophage and neutrophil phenotypes are dependent on the type of expressed receptors, the duality of platelet functions is dependent on the strength of the CD40L-related signal: with a weak signal promoting tumour growth but a strong signal inducing cancer cell apoptosis^32^. In obesity, sCD40L is higher^33,34^ and is correlated inversely with serum adiponectin, which blocks sCD40L release from platelets and is lower in obesity^35^. Thus, with low adiponectin levels in obesity, the CD40L-related platelet functions may be shifted from pro-oncogenic to anti-neoplastic and may thus be hindering the positive association of platelets with lung cancer risk. A potential obesity-related modification of the CD40-CD40L system merits further investigation because the latter is more broadly involved in the regulation of immune responses and there is already interest in developing an agonist anti-CD40 antibody as a promising cancer treatment strategy targeting the apoptosis of CD40-expressing tumours^36^.

Using the allometric AFI and ALI, we have shown that the previously described positive association of FM with lung cancer risk, the lack of association with FM in women, and the stronger inverse associations with FFM in both sexes^37,38^, are biases resulting from mutual adjustment of anthropometric measures reflecting similar biological traits. Notably, FFM is partly related to obesity since the muscles are a major glycogen storage depot^39^ and both lean and fat mass increase after overfeeding^40^. Although the inverse association of BMI with lung cancer risk is well known and is observed only in smokers (current or former)^7,8^, the inverse associations of ALI and AFI with lung cancer risk would not be explained simply by residual confounding by smoking, as they were influenced little by a detailed adjustment for smoking status and intensity and were similar in non-smokers (never and former) and in current smokers (for ALI in men and for AFI in women). It is unlikely that they would be entirely accounted for by reverse causality either, as they were retained for longer follow-up in women and were only partly attenuated in men. Given that in never smokers genetically predicted BMI has been inversely associated with lung cancer risk overall and with lung adenocarcinomas but positively associated with squamous cell and small cell carcinomas of the lung^41^, future investigations would need to clarify the mechanisms underlying the associations of body composition with lung cancer risk and any heterogeneity by sex and lung cancer histology.

Surprisingly little is known about prospective associations of transaminases with lung cancer risk. To our knowledge, our study is the first to show an inverse association with ALT but not AST mainly in men when a smaller-size study has recently found no evidence for association^42^. An inverse association with ALT is compatible with our previously presented argument that obesity-related NAFLD and liver fibrosis contribute to reduction of platelet count and hence a lower lung cancer risk in obesity^3^, a process potentially hindered in women because oestrogens are associated with lower risk of NAFLD^9^. The positive associations with GGT and ALP, which agree with the findings of other prospective studies^42–44^, may be stronger in men than in women because men are more likely to smoke and to consume more alcohol, but only the positive association with ALP in men was retained for longer follow-up time (potentially reflecting lower liver clearance of smoking related or environmental carcinogens), while a reverse causality, with high ALP due to liver and bone metastases near diagnosis, is more likely in women^45,46^. Neither GGT nor ALP showed interactions with platelet parameters, so it is unlikely that their associations with lung cancer risk are related to platelets. BLT, which has anti-inflammatory and antioxidant properties^47^, also appeared unrelated to platelets. Although BLT was inversely associated with lung cancer risk in men, in agreement with previous observational and genetic studies^48–50^, it did not show interactions with platelet parameters.

Strengths of our study are the prospective cohort design with a reasonably large number of incident lung cancer cases and available BIA body composition measurements, liver function tests, and platelet parameters for most of the cohort, which enabled us to examine interactions. We derived allometric body composition indices, which define fat and lean mass independent of each other and of height. We were also able to adjust for major lifestyle factors and, most importantly for detailed categories of smoking intensity and duration of smoking cessation, thus minimising confounding.

Clear limitations of our study are the lack of information about platelet-derived and obesity-related factors (such as adipokines), or information about air pollution. Body composition measurements were limited to BIA, as more accurate imaging measurements were only available for a smaller part of the UK Biobank cohort and were obtained several years after baseline, hence with a shorter follow-up time. Since ALT is within the normal range in about a quarter of patients with NAFLD^51^, ALT as a proxy of NAFLD may be underestimating liver fat infiltration but imaging measurements of liver fat infiltration were also available only for a limited part of the cohort. A larger number of lung cancer cases would be necessary to examine heterogeneity in the interactions with platelet parameters according to lung cancer histology. The number of lung cancer cases in non-smokers was limited, preventing separate examination of never smokers. We could not consider changes in the exposures and the confounders during follow-up, as only a baseline measurement was available for most participants, or ethnic differences, as the larger part of UK Biobank participants have white ethnic background. Their lifestyle is also healthier compared to the general population^52^, which may have limited the variability in body composition and liver function. Last, although we have examined prospective associations and interactions and have proposed potential mechanistic explanations of our findings, our observations cannot directly be interpreted as causal and further investigations would be required to strengthen the support for causality.

## Conclusions

Only high-AFI and high-ALT attenuated the positive association of PLT and the inverse association of MPV with lung cancer risk in men in the same way as BMI, suggesting the involvement of an adiposity-related factor in a sex-specific manner. Investigating further the attenuation of the pro-oncogenic associations with platelets in obesity could potentially reveal new approaches to lung cancer treatment.

## Electronic supplementary material

Below is the link to the electronic supplementary material.

Supplementary Material 1

## Data Availability

The dataset analysed in the current study was used under license and cannot be made freely available in a public repository or obtained from the authors due to restrictions related to privacy regulations and informed consent of the participants. Access to the data, however, can be obtained by bona fide researchers from UK Biobank, subject to approval of the research project and a material transfer agreement. For information on how to gain access to UK Biobank data, please follow the instructions at https://www.ukbiobank.ac.uk/enable-your-research Further queries related to the data could be addressed to the corresponding author Dr Sofia Christakoudi s.christakoudi@imperial.ac.uk.

## Abbreviations

AFI: Allometric fat–mass index
ALI: Allometric lean–mass index
ALP: Alkaline phosphatase
ALT: Alanine aminotransferase
AST: Aspartate aminotransferase
BLD: Direct (conjugated) bilirubin
BLT: Total bilirubin
BMI: Body mass index
CI: Confidence interval
FFM: Total fat–free mass (bioelectrical impedance measurement)
FM: Total fat mass (bioelectrical impedance measurement)
GGT: Gamma–glutamyl transferase
HR: Hazard ratio
HRT: Hormone replacement therapy
ICD10: 10th revision of the International Statistical Classification of Diseases
MPV: Mean platelet volume
PLT: Platelet count
RERI: Relative excess risk from interaction (additive interaction)
SD: Standard deviation
UK: United Kingdom
