# Supplementary material for: Allometric fat mass index and alanine aminotransferase attenuate the associations of platelet parameters with lung cancer risk
Source: Sci Rep. 2024 Nov 1;14:26318. doi: 10.1038/s41598-024-78281-x (PMC11530616; doi:10.1038/s41598-024-78281-x)
Supplement: Supplementary file 1 — Supplementary Material 1 [file 41598_2024_78281_MOESM1_ESM.pdf]

Allometric fat mass index and alanine aminotransferase attenuate the associations of platelet parameters with lung cancer risk

Sofia Christakoudi, Konstantinos K. Tsilidis, Marc J. Gunter, Elio Riboli

Supplementary Tables

Supplementary Table S1 Flow chart of study participants 2
Supplementary Table S2 Derivation of allometric body composition indices 3
Supplementary Table S3 Imputation of undetected liver function tests 4
Supplementary Table S4 Summaries for the dataset with all available liver function tests 5
Supplementary Table S5 Anthropometric indices, platelet parameters, and liver function tests in cross-classification groups (men) 6

Supplementary Figures

Supplementary Figure S1 Pairwise correlations between body composition, platelet parameters, and liver function tests 8
Supplementary Figure S2 Pairwise associations of non-smoking candidate covariates with exposures and lung cancer risk 9
Supplementary Figure S3 Associations with lung cancer risk: comparisons between allometric and traditional body composition indices 11
Supplementary Figure S4 Associations of liver function tests with lung cancer risk and multiplicative interactions with platelet parameters 12
Supplementary Figure S5 Associations of anthropometric indices and liver function tests with lung cancer risk: adjustment for platelet parameters........ 13
Supplementary Figure S6 Associations of anthropometric indices and liver function tests with lung cancer risk: sensitivity analyses 14
Supplementary Figure S7 Three-way cross-classifications according to sex, platelet parameters, and one of BMI, AFI, or ALT 16
Supplementary Figure S8 Interactions with platelet parameters: sensitivity analyses (men) 17
References 19

Supplementary Table S1 Flow chart of study participants from the UK Biobank cohort

	**Exclusions**	**Men**	**Women**
	Total available (excluding consent withdrawals by the time of analysis):	229,054	273,279
1.	Ethnic background (restricted to self-reported white) [a]	13,861	15,932
2.	Anthropometric measurements missing or extreme [b]	2372	4758
3.	Sex mismatch (genetic & self-reported); sex chromosome aneuploidy; age <40 or >70 years; pregnant at recruitment [a]	405	493
4.	Prevalent cancer at recruitment [a]	12,188	21,854
5.	Antihemorrhagic agents [c]	14	582
6.	Platelet parameters – any missing	7869	10,914
7.	Bioelectrical impedance or liver function measurements − all missing	124	100
	Total excluded (% from total available with active consent):	36,833 (16.1)	54,633 (20.0)
	**Total included in the study [d]**	**192,221**	**218,646**
	Body composition measurements − all available (% total in study)	188,946 (98.3)	216,101 (98.8)
	Liver function test measurements − all available (% total in study)	183,104 (95.3)	208,339 (95.3)
	Body composition & liver function tests – all available	179,883	205,819
	(% body composition dataset / % liver function tests dataset)	(95.2 / 98.8)	(95.2 / 98.2)

The exclusion criteria were applied sequentially in the displayed order, counting each excluded individual only once.

[a] – for UK Biobank Field names, definition of variables, and definition of prevalent cancer cases see Supplementary Methods in [15].

[b] – missing anthropometric measurements; height <130 cm; waist circumference <50 or >160 cm; body mass index (BMI) <18.5 or ≥45 kg/m^2. Field names for waist and hip circumferences, weight, and height are listed in Supplementary Methods of [15].

[c] – self-reported use of medications from Fields [20003-0/47] “Treatment/ medication code” with the following codes: 1140861766 (ethamsylate), 1140861832 (tranexamic acid), 1140861834 (cyklokapron 500mg tablet).

[d] – note that some participants contributed only to the complete body composition subset and others only to the complete liver function tests subset, but all participants included in the study contributed to at least one of the two complete subsets, to the definition of the median sex-specific categories of covariates (used for imputation of missing values), and to the definition of sex-specific tertiles or median and z-scores of body mass index and platelet parameters; all participants with available measurements for any of the body composition or liver function test variables contributed to the definition of sex-specific tertiles and z-scores.

Supplementary Table S2 Derivation of allometric body composition indices

	Intercept	Height	FM	R^2
**AFI** $_{men}$	2.4574 (0.0122)	1.0290 (0.0217)		0.012
**AFI** $_{women}$	2.5632 (0.0095)	1.3582 (0.0195)		0.022
**ALI** $_{men}$	2.6780 (0.0027)	1.8122 (0.0044)	0.1481 (0.0005)	0.614
**ALI** $_{women}$	2.6709 (0.0022)	1.1960 (0.0039)	0.1670 (0.0004)	0.575

AFI – allometric fat-mass index; **ALI** – allometric lean-mass index; **BIA** – bioelectrical impedance; **FM** – total (whole body) fat mass (BIA measurement) [Field 23100-2.0]; **FFM** – total (whole body) fat-free mass (BIA measurement) [Field 23101-2.0]; $\mathbf{R^2}$ – proportion explained variability.

Linear regression models (sex-specific) for AFI:

$$\ln(\mathbf{FM}, \text{kg}) \sim \ln(\mathbf{Height}, \text{m})$$

Linear regression models (sex-specific) for ALI:

$$\ln(\mathbf{FFM}, \text{kg}) \sim \ln(\mathbf{Height}, \text{m}) + \ln(\mathbf{FM}, \text{kg})$$

AFI and ALI could be calculated as shown in the main document (using only multiplication and minus sign for the power coefficients of height and FM), or with division as shown below:

$$AFI_{men} = \frac{\text{FM (kg)}}{\text{Height (m)}^{\,1.0290}}$$

$$AFI_{women} = \frac{\text{FM (kg)}}{\text{Height (m)}^{\,1.3582}}$$

$$ALI_{men} = \frac{\text{FFM (kg)}}{\text{Height (m)}^{\,1.8122} * \text{FM (kg)}^{\,0.1481}}$$

$$ALI_{women} = \frac{\text{FFM (kg)}}{\text{Height (m)}^{\,1.1960} * \text{FM (kg)}^{\,0.1670}}$$

The use of allometric body composition indices instead of traditional body composition measures was determined by two reasons. Similarly to BMI, scaling for body size reflected in height was necessary to account for the larger average size of all body components in larger individuals. Deriving the scaling power coefficients for height separately for FM and FFM and separately in men and women was necessary because these differed from the power two coefficient in the formula for BMI, indicating a different relationship of height with weight overall and with its individual components FM and FFM.

Notably, the scaling of FFM for height factors out bone mass, which is included in FFM measured with BIA (hence the strong positive correlation of FFM with height). Further scaling of FFM for FM was necessary because BIA quantifies FFM indirectly, based on the difference in electrical conductivity between body water and body fat and assuming constant hydration, while hydration is higher in obesity [17] and FFM is overestimated in obesity [18]. In addition, both lean and fat mass increase after overfeeding [40] as the muscles are a major glycogen storage depot [39]. Thus, the scaling of FFM for FM would factor out any components in FFM related to muscle mass as an indicator of altered energy balance and storage, confining this role to FM and defining ALI as an index of lean mass.

Supplementary Table S3 Imputation of undetected liver function tests

	Field $_{\text{Value}}$	Field $_{\text{QC}}$	Min	Low	Max	High
**ALT**	30620-0.0	30626-0.0	3.01	34	495.19	17
**AST**	30650-0.0	30656-0.0	3.30	<10	947.20	<10
**GGT**	30730-0.0	30736-0.0	5.00	15	1165.90	55
**ALP**	30610-0.0	30616-0.0	8.00	<10	1231.10	<10
**BLD**	30660-0.0	30666-0.0	1.00	55,588 [#]	70.06	<10
**BLT**	30840-0.0	30846-0.0	1.08	10	144.52	<10

ALP – alkaline phosphatase; **ALT** – alanine aminotransferase; **AST** – aspartate aminotransferase; **BLD** – direct (conjugated) bilirubin; **BLT** – total bilirubin; **Field $_{\text{QC}}$** – biomarker reportability field; **Field $_{\text{Value}}$** – biomarker value field; **GGT** – gamma-glutamyl transferase; **High** – number of participants with attempted measurements above the upper limit of detection; **Low** – number of participants with attempted measurements below the lower limit of detection ([#] 13.5% of total with attempted measurements); **Max** – highest detected value; **Min** – lowest detected value.

Reportability codes:

- **1**: "Reportable at assay and after aliquot correction, if attempted" – values used as provided.
- **2**: "Reportable at assay but not reportable after any corrections (too low)" – as for code 4.
- **3**: "Reportable at assay but not reportable after any corrections (too high)" – as for code 5.
- **4**: "Not reportable at assay (too low)" – replaced with half the lowest detected level.
- **5**: "Not reportable at assay (too high)" – replaced with the highest detected level.

Direct bilirubin was imputed with quantile regression imputation of truncated left-censored data (QRILC) (imputeLCMD v2.0 package in R) [21], which uses the available data to estimate the parameters of the distribution (tunning parameter sigma=1).

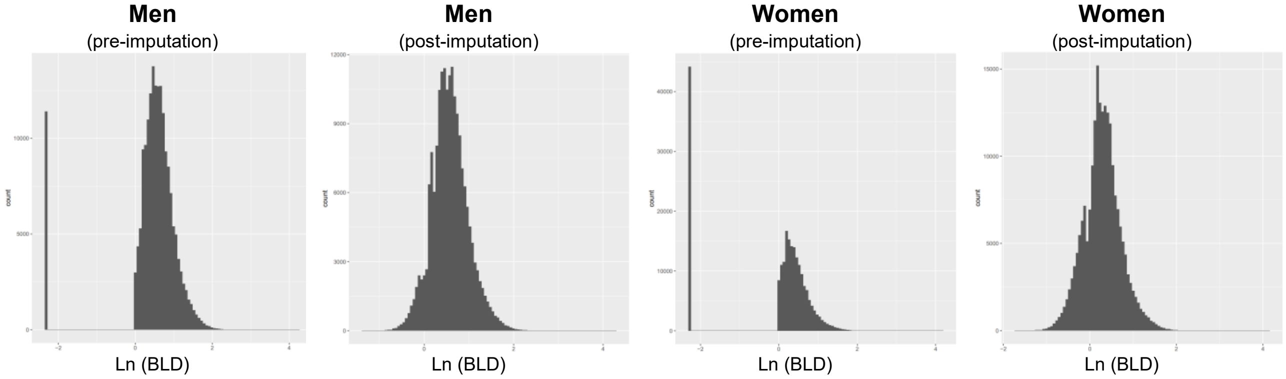

Values for participants without attempted measurements were considered missing.

Supplementary Table S4 Summaries for the dataset with all available liver function tests

	Total	Never smokers	Former smokers	Current smokers	p
**MEN**					
Cohort: n (%)	183,104	89,052 (48.6)	71,792 (39.2)	22,260 (12.2)	
Cases: n (rate)	1541 (819)	130 (140)	755 (1038)	656 (2920)	
Time to diagnosis [a]	6.4 (3.4−8.9)	6.4 (3.1−8.8)	6.4 (3.4−9.0)	6.3 (3.6−8.8)	0.894
Age (years) [b]	57.2 (8.1)	56.0 (8.2)	59.3 (7.6)	55.5 (8.2)	$8*10^{-179}$
Height (cm) [b]	175.9 (6.8)	176.2 (6.8)	175.7 (6.7)	175.5 (6.8)	$5*10^{-64}$
BMI (kg/m^2) [b]	27.8 (4.0)	27.4 (3.9)	28.4 (4.0)	27.4 (4.2)	$5*10^{-78}$
PLT (*10^9/L) [c]	232 (146−370)	231 (146−365)	232 (145−370)	239 (147−389)	$2*10^{-72}$
MPV (fL) [c]	9.22 (7.41−11.49)	9.22 (7.41−11.46)	9.23 (7.39−11.52)	9.25 (7.41−11.53)	$5*10^{-5}$
ALT (IU/L) [c]	24.8 (10.5−58.4)	24.7 (10.6−57.6)	25.3 (10.8−59.3)	23.5 (9.5−58.2)	$2*10^{-10}$
AST (IU/L) [c]	27.0 (15.7−46.3)	27.0 (16.0−45.3)	27.4 (15.9−47.0)	25.8 (14.1−47.4)	$2*10^{-29}$
GGT (IU/L) [c]	36.2 (11.1−118.5)	33.9 (10.7−107.4)	38.2 (11.6−125.4)	39.5 (11.3−137.8)	$<1*10^{-311}$
ALP (IU/L) [c]	78.9 (47.4−131.2)	77.7 (47.2−127.9)	78.7 (47.1−131.3)	84.5 (50.5−141.7)	$1*10^{-311}$
BLD (μmol/L) [c]	1.78 (0.8−3.95)	1.83 (0.83−4.05)	1.79 (0.82−3.93)	1.55 (0.70−3.43)	$<1*10^{-311}$
BLT (μmol/L) [c]	9.5 (4.5−20.2)	9.9 (4.6−21.0)	9.5 (4.6−19.8)	8.3 (4.1−16.8)	$<1*10^{-311}$
**WOMEN (liver function tests)**					
Cohort: n (%)	208,339	123,208 (59.1)	66,882 (32.1)	18,249 (8.8)	
Cases: n (rate)	1428 (652)	263 (202)	617 (883)	548 (2898)	
Time to diagnosis [a]	6.6 (4.0−9.0)	6.4 (3.7−9.2)	6.5 (3.9−8.9)	6.8 (4.3−9.1)	0.572
Age (years) [b]	56.9 (8.0)	56.6 (8.0)	57.9 (7.7)	54.8 (8.0)	$3*10^{-4}$
Height (cm) [b]	162.6 (6.2)	162.6 (6.2)	162.8 (6.2)	162.5 (6.3)	0.007
BMI (kg/m^2) [b]	26.9 (4.8)	26.8 (4.8)	27.2 (4.8)	26.7 (4.7)	$5*10^{-19}$
PLT (*10^9/L) [c]	260 (165−409)	259 (165−407)	260 (165−409)	264 (164−425)	$3*10^{-18}$
MPV (fL) [c]	9.31 (7.46−11.62)	9.30 (7.45−11.60)	9.30 (7.45−11.61)	9.38 (7.49−11.76)	$3*10^{-14}$
ALT (IU/L) [c]	18.2 (7.9−41.8)	18.1 (7.9−41.4)	18.7 (8.1−43.1)	17.2 (7.5−39.7)	0.001
AST (IU/L) [c]	23.5 (14.1−39.1)	23.5 (14.2−38.8)	23.8 (14.2−40.0)	22.2 (13.1−37.5)	$1*10^{-37}$
GGT (IU/L) [c]	23.8 (7.6−74.4)	23.2 (7.5−71.4)	24.6 (7.8−77.9)	25.5 (8.0−80.9)	$7*10^{-161}$
ALP (IU/L) [c]	80.9 (45.7−143.1)	80.4 (45.5−142.0)	81.1 (45.9−143.3)	83.3 (46.6−148.8)	$7*10^{-51}$
BLD (μmol/L) [c]	1.33 (0.58−3.08)	1.35 (0.58−3.13)	1.34 (0.59−3.06)	1.19 (0.53−2.68)	$1*10^{-187}$
BLT (μmol/L) [c]	7.6 (3.7−15.6)	7.7 (3.7−15.9)	7.6 (3.7−15.5)	6.8 (3.4−13.4)	$2*10^{-254}$

ALP – alkaline phosphatase; **ALT** – alanine aminotransferase; **AST** – aspartate aminotransferase; **BLD** – direct (conjugated) bilirubin; **BLT** – total bilirubin; **BMI** – body mass index; **GGT** – gamma-glutamyl transferase; **MPV** – mean platelet volume; **n (%)** – number of participants per group (percentage from total per sex); **n (rate)** – number of lung cancer cases per group (incidence rate per $1*10^6$ person years); **PLT** – platelet count.

[a] median (interquartile range: 25th-75th centile); [b] mean (standard deviation); [c] geometric mean (95% reference range).

Smoking status groups per sex were compared with analysis of variance (after log-transformation for biomarkers).

Supplementary Table S5 Anthropometric indices, platelet parameters, and liver function tests in cross-classification groups (men)

MEN								
	**Low−PLT Low−BMI**	**High−PLT Low−BMI**	**Low−PLT High−BMI**	**High−PLT High−BMI**	**Low−MPV Low−BMI**	**High−MPV Low−BMI**	**Low−MPV High−BMI**	**High−MPV High−BMI**
Cohort: n (%)	62,018 (32.8)	63,911 (33.8)	31,900 (16.9)	31,117 (16.5)	63,973 (33.9)	61,956 (32.8)	30,338 (16.1)	32,679 (17.3)
Cases: n (rate)	392 (618)	631 (951)	294 (913)	256 (797)	561 (853)	462 (722)	238 (768)	312 (936)
Age (years) [a]	57.7 (8.2)	56.5 (8.2)	58.3 (7.8)	56.5 (8.0)	57.2 (8.1)	57.0 (8.3)	57.3 (7.9)	57.5 (7.9)
BMI (kg/m^2) [a]	25.6 (2.2)	25.5 (2.2)	32.3 (3.0)	32.3 (3.0)	25.5 (2.2)	25.6 (2.2)	32.2 (2.9)	32.4 (3.0)
AFI [a]	10.1 (2.6)	10.2 (2.6)	16.9 (3.6)	16.9 (3.6)	10.2 (2.6)	10.2 (2.6)	16.9 (3.6)	17.0 (3.7)
ALI [a]	14.4 (1.0)	14.3 (1.0)	15.1 (1.0)	15.1 (0.9)	14.3 (1.0)	14.4 (1.0)	15.1 (0.9)	15.1 (0.9)
PLT (*10^9/L) [b]	195 (139−273)	277 (212−363)	193 (133−279)	276 (214−358)	254 (167−388)	213 (138−329)	252 (164−387)	212 (134−335)
MPV (fL) [b]	9.6 (7.8−11.9)	8.8 (7.3−10.6)	9.7 (7.8−12.1)	8.9 (7.4−10.7)	8.4 (7.5−9.5)	10.1 (8.7−11.7)	8.5 (7.5−9.5)	10.1 (8.7−11.8)
ALT (IU/L) [b]	22.8 (10.1−51.1)	22.7 (10.3−50.2)	29.6 (12.2−71.7)	29.2 (12.4−68.7)	22.6 (10.2−50.4)	22.9 (10.3−50.9)	29.1 (12.3−69.1)	29.6 (12.3−71.2)
	**Low−PLT Low−AFI**	**High−PLT Low−AFI**	**Low−PLT High−AFI**	**High−PLT High−AFI**	**Low−MPV Low−AFI**	**High−MPV Low−AFI**	**Low−MPV High−AFI**	**High−MPV High−AFI**
Cohort: n (%)	62,230 (32.9)	63,721 (33.7)	31,688 (16.8)	31,307 (16.6)	63,899 (33.8)	62,052 (32.8)	30,412 (16.1)	32,583 (17.2)
Cases: n (rate)	367 (575)	579 (873)	319 (1001)	308 (959)	516 (783)	430 (669)	283 (916)	344 (1040)
Age (years) [a]	57.3 (8.2)	56.1 (8.2)	59.1 (7.5)	57.3 (7.9)	56.8 (8.2)	56.6 (8.3)	58.2 (7.7)	58.2 (7.8)
BMI (kg/m^2) [a]	25.8 (2.4)	25.7 (2.4)	32.0 (3.3)	31.9 (3.3)	25.7 (2.4)	25.8 (2.4)	31.9 (3.3)	32.1 (3.3)
AFI [a]	10.0 (2.4)	10.0 (2.3)	17.3 (3.3)	17.3 (3.3)	10.0 (2.4)	10.0 (2.4)	17.2 (3.2)	17.4 (3.3)
ALI [a]	14.6 (1.1)	14.5 (1.1)	14.8 (1.0)	14.7 (1.0)	14.5 (1.1)	14.6 (1.1)	14.7 (1.0)	14.8 (1.0)
PLT (*10^9/L) [b]	195 (139−273)	277 (212−363)	193 (133−280)	277 (213−359)	254 (167−387)	213 (138−329)	253 (164−389)	212 (134−336)
MPV (fL) [b]	9.6 (7.8−11.9)	8.8 (7.3−10.6)	9.7 (7.8−12.1)	8.9 (7.3−10.7)	8.4 (7.5−9.5)	10.1 (8.7−11.7)	8.5 (7.5−9.5)	10.1 (8.7−11.8)
ALT (IU/L) [b]	22.8 (10.2−51.3)	22.8 (10.3−50.4)	29.5 (12.1−71.7)	29.0 (12.3−68.3)	22.7 (10.2−50.5)	23.0 (10.3−51.2)	29.0 (12.1−69.1)	29.5 (12.2−70.9)
	**Low−PLT Low−ALT**	**High−PLT Low−ALT**	**Low−PLT High−ALT**	**High−PLT High−ALT**	**Low−MPV Low−ALT**	**High−MPV Low−ALT**	**Low−MPV High−ALT**	**High−MPV High−ALT**
Cohort: n (%)	60,458 (33.0)	61,571 (33.6)	30,586 (16.7)	30,489 (16.7)	61,442 (33.6)	60,587 (33.1)	29,892 (16.3)	31,183 (17.0)
Cases: n (rate)	455 (742)	698 (1101)	206 (656)	182 (569)	620 (989)	533 (859)	175 (564)	213 (658)
Age (years) [a]	58.6 (8.0)	57.4 (8.0)	56.6 (8.0)	54.7 (8.0)	58.1 (8.0)	57.9 (8.1)	55.6 (8.0)	55.8 (8.1)
BMI (kg/m^2) [a]	27.0 (3.8)	26.9 (3.8)	29.5 (4.1)	29.3 (4.1)	26.9 (3.7)	27.1 (3.8)	29.3 (4.0)	29.5 (4.1)
AFI [a]	11.6 (4.1)	11.6 (4.1)	14.2 (4.4)	14.1 (4.3)	11.5 (4.0)	11.7 (4.2)	14.0 (4.3)	14.2 (4.4)
ALI [a]	14.6 (1.1)	14.5 (1.1)	14.7 (1.1)	14.7 (1.1)	14.5 (1.1)	14.6 (1.1)	14.7 (1.1)	14.7 (1.1)
PLT (*10^9/L) [b]	195 (139−273)	277 (212−362)	193 (135−277)	277 (213−360)	254 (166−386)	213 (138−330)	253 (165−389)	212 (135−332)
MPV (fL) [b]	9.6 (7.8−12.0)	8.8 (7.3−10.6)	9.7 (7.8−12.0)	8.8 (7.3−10.7)	8.4 (7.5−9.5)	10.1 (8.7−11.7)	8.5 (7.5−9.5)	10.1 (8.7−11.8)
ALT (IU/L) [b]	19.5 (11.6−32.7)	19.5 (11.6−32.7)	40.3 (21.9−74.1)	39.6 (22.5−69.9)	19.4 (11.5−32.7)	19.6 (11.7−32.7)	39.7 (22.3−70.7)	40.2 (22.1−73.2)

AFI – allometric fat-mass index (cut-off: ≥13.703); **ALI** – allometric lean-mass index; **ALT** – alanine aminotransferase (cut-off: ≥28.65 IU/L); **BMI** – body mass index (cut-off: ≥28.982 kg/m^2); **MPV** – mean platelet volume (cut-off: ≥9.17 fL); **n (%)** – number of participants per group (percentage from total in men); **n (rate)** – number of lung cancer cases per group (incidence rate per $1*10^6$ person years); **PLT** – platelet count (cut-off: ≥$234.0*10^9$/L).

[a] mean (standard deviation); [b] geometric mean (95% reference range).

Low/High for cross-classifications in men were defined with respect to ≥ median (sex-specific) for PLT and MPV and ≥upper tertile cut-off (sex-specific) for BMI, AFI, and ALT.

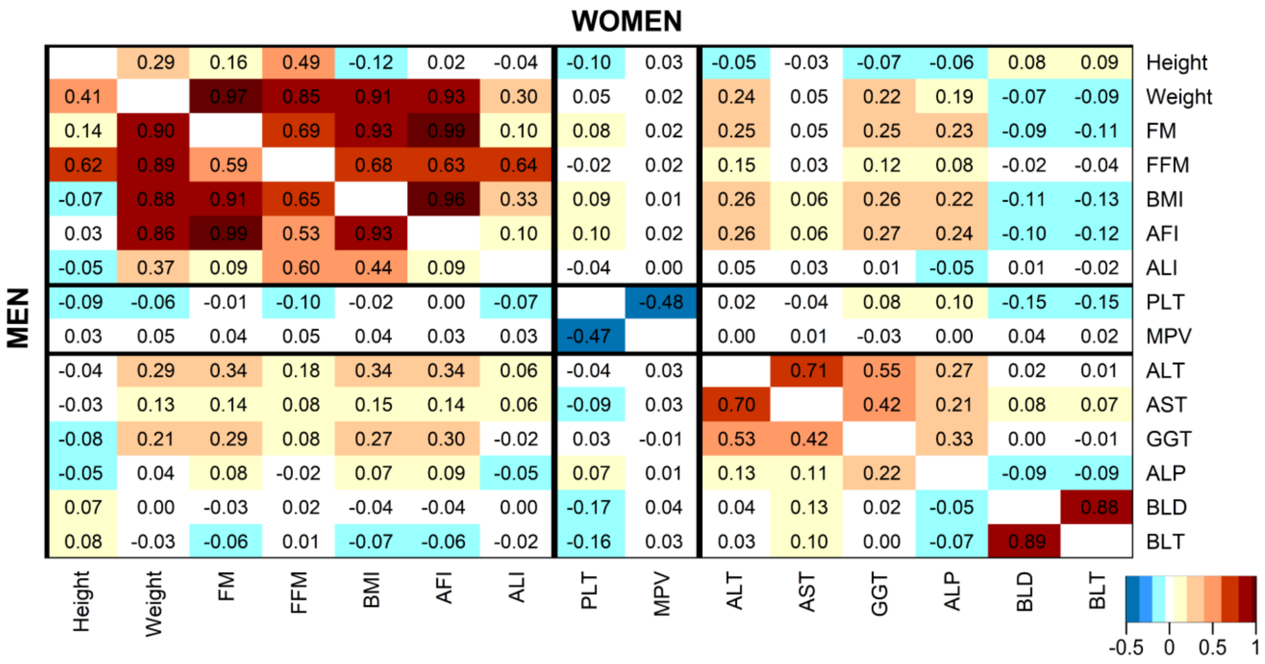

Supplementary Figure S1 Pairwise correlations between body composition, platelet parameters, and liver function tests

AFI – allometric fat-mass index; **ALI** – allometric lean-mass index; **ALP** – alkaline phosphatase; **ALT** – alanine aminotransferase; **AST** – aspartate aminotransferase; **BLD** – direct (conjugated) bilirubin; **BLT** – total bilirubin; **BMI** – body mass index; **FFM** – total fat-free mass (bioelectrical impedance measurement); **FM** – total fat mass (bioelectrical impedance measurement); **GGT** – gamma-glutamyl transferase; **MPV** – mean platelet volume; **PLT** – platelet count.

Partial Pearson correlation coefficients adjusted for age at recruitment were calculated in a subset with available all body composition measurements and all liver function tests (179,883 men; 205,819 women).

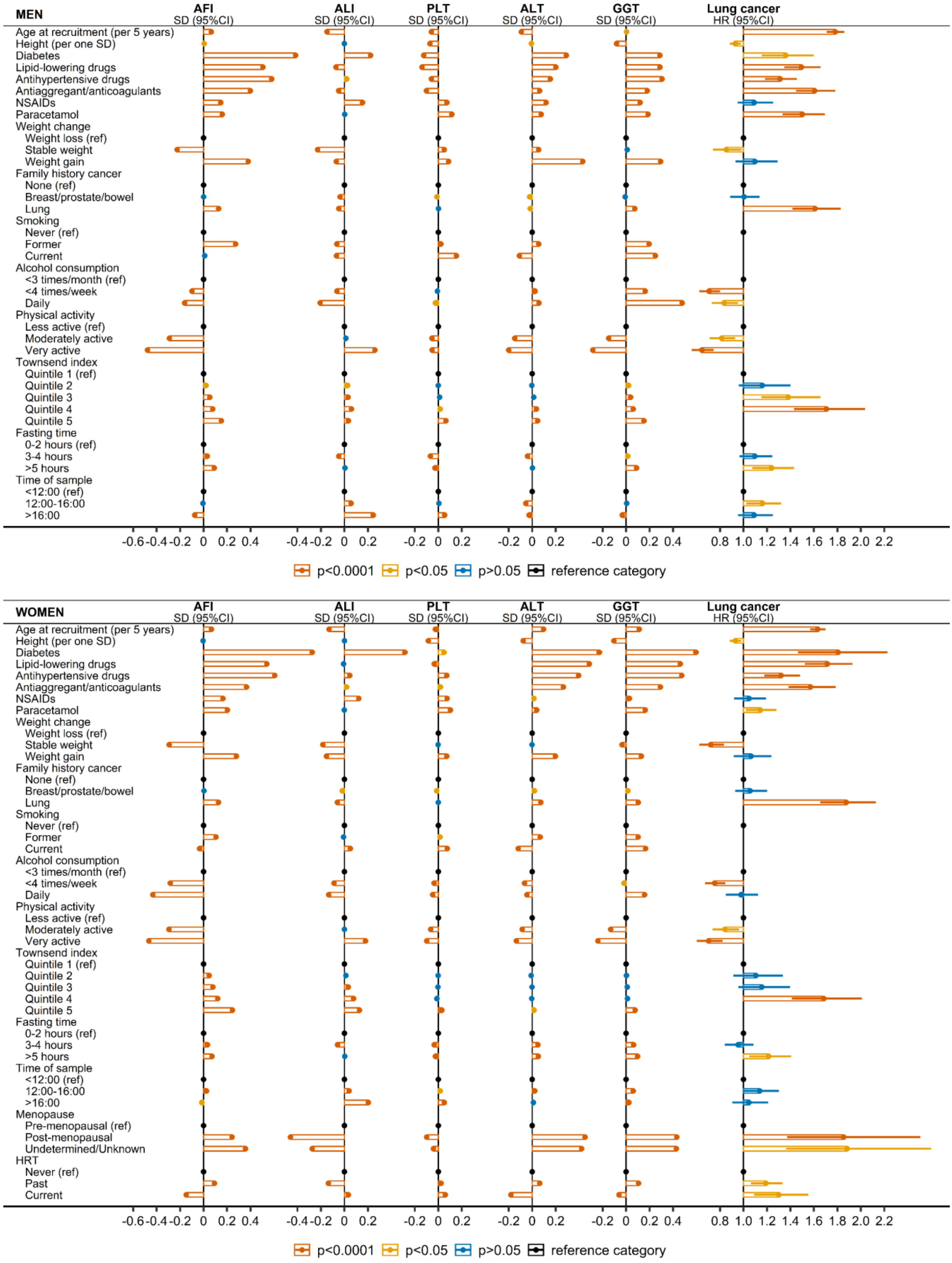

Supplementary Figure S2 Pairwise associations of non-smoking candidate covariates with exposures and lung cancer risk

AFI – allometric fat-mass index; **ALI** – allometric lean-mass index; **ALT** – alanine aminotransferase; **CI** – confidence interval; **GGT** – gamma-glutamyl transferase; **HR** – hazard ratio; **HRT** – hormone replacement therapy; **NSAIDs** – non-steroidal anti-inflammatory drugs; **PLT** – platelet count; **SD** – standard deviation.

Estimates from liner regression models (SD) including individually each body composition index, platelet parameter, or liver function test specified in the header as exposure on a continuous scale (sex-specific z-scores, mean minus value divided by SD, following log-transformation for biomarkers) or Cox proportional hazards models (HR) with timescale age at recruitment (except for age at recruitment as exposure, for which timescale was person years of follow-up). Sex-specific models included each potential candidate covariate individually as the independent variable. Associations with HRT were examined in a subset excluding pre-menopausal women, as very few of them had used HRT.

The following covariates were defined as previously described in reference [15]: region of the assessment centre, weight change within the year preceding recruitment (as an indicator of weight dynamics), smoking status (in this study former smoker included former occasional and former regular smoker), alcohol consumption, physical activity, family history of cancer (in parents or siblings), and Townsend deprivation index (proxy of socio-economic status, quintiles used in this study). The following covariates were defined as previously described in reference [53]: time of blood collection, fasting time, self-reported diabetes (no/yes, including use of antidiabetic drugs and assuming that all participants with self-reported diabetes were treated), use of lipid lowering drugs (no/yes, additionally including cholestyramine products for this study), use of antihypertensive drugs (no/yes), paracetamol use (no/yes), and menopausal status. Use of antiaggregant/anticoagulants (no/yes) and NSAIDs (no/yes) were defined according to reference [3]. HRT use was defined similarly to reference [54].

Although **NSAIDs** use was associated positively with the exposures, there was little evidence for association with lung cancer risk, so this would not be a confounder and was thus omitted. We consolidated weight loss and stable weight in one category and used a binary variable for recent **weight gain** (no/yes) in the final analyses because recent weight loss could reflect reverse causality from lung cancer and could thus act as a collider. We also used a binary variable for **family history of lung cancer** because family history of breast, prostate, or bowel cancer was not associated with the exposures or with lung cancer risk. The remaining candidate covariates were associated to some extend with the exposures and with lung cancer risk and were retained in the final fully adjusted models. A combined variable of smoking status and intensity, defined as in reference [3], was used for model stratification (never smoked; just tried; former occasional; former regular quit≥20 years; former regular quit≥10 years; former regular quit<10 years; current occasional; current regular ≤10 cigarettes/day; current regular >10 cigarettes/day). A combined variable of menopausal status and HRT use, defined as in reference [3], was used for adjustment in women (pre-menopausal; post/unknown menopause never HRT; post/unknown menopause past HRT; post/unknown menopause current HRT).

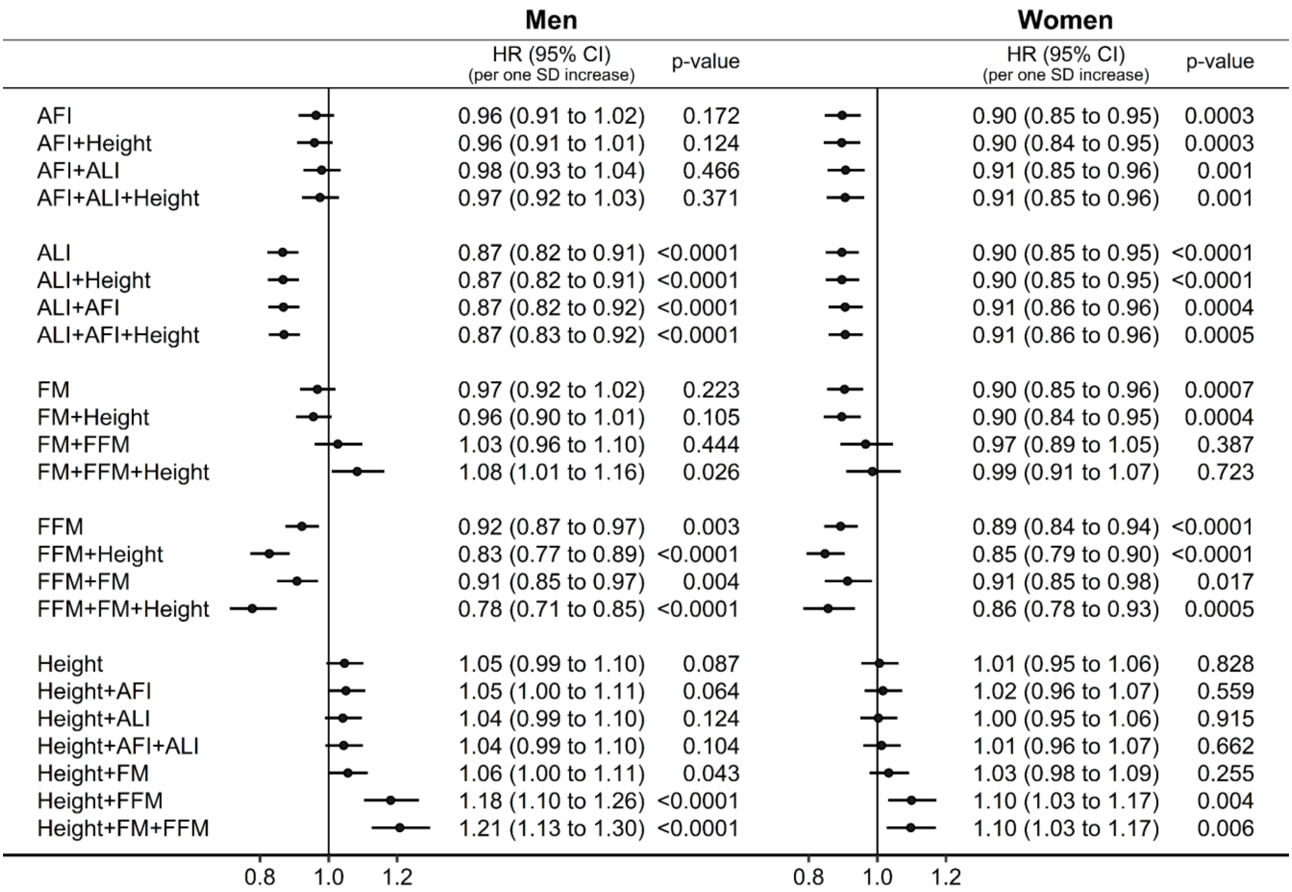

	Men HR (95% CI) (per one SD increase)	Men p-value	Women HR (95% CI) (per one SD increase)	Women p-value
AFI	0.96 (0.91 to 1.02)	0.172	0.90 (0.85 to 0.95)	0.0003
AFI+Height	0.96 (0.91 to 1.01)	0.124	0.90 (0.84 to 0.95)	0.0003
AFI+ALI	0.98 (0.93 to 1.04)	0.466	0.91 (0.85 to 0.96)	0.001
AFI+ALI+Height	0.97 (0.92 to 1.03)	0.371	0.91 (0.85 to 0.96)	0.001
ALI	0.87 (0.82 to 0.91)	<0.0001	0.90 (0.85 to 0.95)	<0.0001
ALI+Height	0.87 (0.82 to 0.91)	<0.0001	0.90 (0.85 to 0.95)	<0.0001
ALI+AFI	0.87 (0.82 to 0.92)	<0.0001	0.91 (0.86 to 0.96)	0.0004
ALI+AFI+Height	0.87 (0.83 to 0.92)	<0.0001	0.91 (0.86 to 0.96)	0.0005
FM	0.97 (0.92 to 1.02)	0.223	0.90 (0.85 to 0.96)	0.0007
FM+Height	0.96 (0.90 to 1.01)	0.105	0.90 (0.84 to 0.95)	0.0004
FM+FFM	1.03 (0.96 to 1.10)	0.444	0.97 (0.89 to 1.05)	0.387
FM+FFM+Height	1.08 (1.01 to 1.16)	0.026	0.99 (0.91 to 1.07)	0.723
FFM	0.92 (0.87 to 0.97)	0.003	0.89 (0.84 to 0.94)	<0.0001
FFM+Height	0.83 (0.77 to 0.89)	<0.0001	0.85 (0.79 to 0.90)	<0.0001
FFM+FM	0.91 (0.85 to 0.97)	0.004	0.91 (0.85 to 0.98)	0.017
FFM+FM+Height	0.78 (0.71 to 0.85)	<0.0001	0.86 (0.78 to 0.93)	0.0005
Height	1.05 (0.99 to 1.10)	0.087	1.01 (0.95 to 1.06)	0.828
Height+AFI	1.05 (1.00 to 1.11)	0.064	1.02 (0.96 to 1.07)	0.559
Height+ALI	1.04 (0.99 to 1.10)	0.124	1.00 (0.95 to 1.06)	0.915
Height+AFI+ALI	1.04 (0.99 to 1.10)	0.104	1.01 (0.96 to 1.07)	0.662
Height+FM	1.06 (1.00 to 1.11)	0.043	1.03 (0.98 to 1.09)	0.255
Height+FFM	1.18 (1.10 to 1.26)	<0.0001	1.10 (1.03 to 1.17)	0.004
Height+FM+FFM	1.21 (1.13 to 1.30)	<0.0001	1.10 (1.03 to 1.17)	0.006

Supplementary Figure S3 Associations with lung cancer risk: comparisons between allometric and traditional body composition indices

AFI – allometric fat-mass index; **ALI** – allometric lean-mass index; **FFM** – total fat-free mass (bioelectrical impedance measurement); **FM** – total fat mass (bioelectrical impedance measurement); **CI** – confidence interval; **HR** – hazard ratio; **p-value** – Wald test for the individual term; **SD** – standard deviation.

Cox proportional hazards models including as exposure the first specified anthropometric index in each set (sex-specific z-scores, value minus mean divided by SD), stratified by age at recruitment, region, and smoking status and intensity, and adjusted for the additional anthropometric indices specified after **+…**, as well as for recent weight gain, alcohol consumption, physical activity, Townsend deprivation index, family history of lung cancer, time of blood collection, fasting time, diabetes, and use of lipid-lowering drugs, antihypertensive drugs, antiaggregant/anticoagulants, and paracetamol, and in women, menopausal status, and hormone replacement therapy use.

Notably, the associations of the allometric AFI and ALI with lung cancer risk were not influenced materially by mutual adjustment or by adjustment for height. The association of FM with lung cancer risk, however, was shifted in the positive direction (becoming positive for men and null for women) after adjustment for FFM and height, while mutual adjustment of FFM and height resulted in stronger associations with lung cancer risk for both (inverse for FFM and positive for height).

	Men HR (95% CI) (per one SD increase)	Men p-value	Women HR (95% CI) (per one SD increase)	Women p-value	p_{sex}
**Associations**					
AST	0.95 (0.91 to 1.01)	0.078	0.97 (0.92 to 1.03)	0.336	0.590
ALP	1.19 (1.13 to 1.24)	<0.0001	1.07 (1.01 to 1.13)	0.031	0.007
BLD	0.98 (0.93 to 1.03)	0.365	1.01 (0.95 to 1.07)	0.714	0.376
BLT	0.90 (0.85 to 0.95)	0.0002	0.97 (0.91 to 1.03)	0.273	0.075
**Interactions PLT**					
AST * PLT	0.96 (0.92 to 1.00)	0.039	1.01 (0.96 to 1.06)	0.800	
ALP * PLT	1.01 (0.97 to 1.06)	0.585	1.03 (0.97 to 1.08)	0.312	
BLD * PLT	1.02 (0.97 to 1.07)	0.360	0.97 (0.92 to 1.02)	0.243	
BLT * PLT	1.02 (0.97 to 1.07)	0.461	0.97 (0.92 to 1.02)	0.190	
**Interactions MPV**					
AST * MPV	1.02 (0.97 to 1.07)	0.431	0.96 (0.91 to 1.01)	0.098	
ALP * MPV	0.96 (0.92 to 1.00)	0.080	1.00 (0.95 to 1.05)	0.970	
BLD * MPV	1.00 (0.95 to 1.05)	0.873	1.02 (0.97 to 1.07)	0.461	
BLT * MPV	0.98 (0.93 to 1.04)	0.552	1.04 (0.98 to 1.09)	0.208	

Supplementary Figure S4 Associations of liver function tests with lung cancer risk and multiplicative interactions with platelet parameters

ALP – alkaline phosphatase; **AST** – aspartate aminotransferase; **CI** – confidence interval; **BLD** – direct (conjugated) bilirubin; **BLT** – total bilirubin; **HR** – hazard ratio; **MPV** – mean platelet volume; **PLT** – platelet count; **SD** – standard deviation; **cases** – number of lung cancer cases; **rate** – incidence rate per $1*10^6$ person years; **p-value** – Wald test for the individual term (associations) or for the individual multiplicative interaction term (interactions); $\mathbf{p_{sex}}$ – p-value comparing the association with lung cancer risk between men and women with the augmentation method of Lunn and McNeil [23].

Cox proportional hazards models with exposure each liver function test individually (associations), or additionally including a multiplicative interaction term between the examined exposure of interest and either PLT or MPV (sex-specific z-scores, value minus mean divided by SD, after log-transformation for biomarkers), stratified by age at recruitment, region, and smoking status and intensity, and adjusted for height, recent weight gain, alcohol consumption, physical activity, Townsend deprivation index, family history of lung cancer, time of blood collection, fasting time, diabetes, and use of lipid-lowering drugs, antihypertensive drugs, antiaggregant/anticoagulants, and paracetamol, and in women, menopausal status and hormone replacement therapy use.

	Men HR (95% CI) (per one SD increase)	Men p-value	Women HR (95% CI) (per one SD increase)	Women p-value
**Anthropometric indices**	cases=1573; rate=810		cases=1473; rate=649	
**BMI**				
Main model	0.91 (0.86 to 0.96)	0.0006	0.87 (0.82 to 0.93)	<0.0001
+ PLT	0.92 (0.87 to 0.97)	0.003	0.87 (0.82 to 0.93)	<0.0001
+ MPV	0.91 (0.86 to 0.96)	0.0010	0.87 (0.82 to 0.93)	<0.0001
**AFI**				
Main model	0.96 (0.91 to 1.01)	0.124	0.90 (0.84 to 0.95)	0.0003
+ PLT	0.97 (0.91 to 1.02)	0.198	0.89 (0.84 to 0.95)	0.0002
+ MPV	0.96 (0.91 to 1.01)	0.148	0.90 (0.84 to 0.95)	0.0003
**ALI**				
Main model	0.87 (0.82 to 0.91)	<0.0001	0.90 (0.85 to 0.95)	<0.0001
+ PLT	0.88 (0.83 to 0.93)	<0.0001	0.90 (0.85 to 0.95)	0.0002
+ MPV	0.87 (0.82 to 0.92)	<0.0001	0.90 (0.85 to 0.95)	<0.0001
**Liver function tests**	cases=1541; rate=819		cases=1428; rate=652	
**ALT**				
Main model	0.90 (0.85 to 0.95)	0.0002	0.95 (0.90 to 1.00)	0.073
+ PLT	0.91 (0.86 to 0.96)	0.0010	0.95 (0.90 to 1.01)	0.086
+ MPV	0.90 (0.86 to 0.96)	0.0003	0.95 (0.90 to 1.00)	0.074
**AST**				
Main model	0.95 (0.91 to 1.01)	0.078	0.97 (0.92 to 1.03)	0.336
+ PLT	0.97 (0.92 to 1.02)	0.265	0.98 (0.93 to 1.03)	0.459
+ MPV	0.97 (0.92 to 1.02)	0.265	0.98 (0.93 to 1.03)	0.459
**GGT**				
Main model	1.06 (1.00 to 1.11)	0.035	1.04 (0.99 to 1.10)	0.110
+ PLT	1.06 (1.01 to 1.12)	0.029	1.04 (0.99 to 1.09)	0.144
+ MPV	1.06 (1.00 to 1.11)	0.037	1.04 (0.99 to 1.10)	0.111
**ALP**				
Main model	1.19 (1.13 to 1.24)	<0.0001	1.07 (1.01 to 1.13)	0.031
+ PLT	1.17 (1.12 to 1.23)	<0.0001	1.06 (1.00 to 1.12)	0.053
+ MPV	1.18 (1.13 to 1.24)	<0.0001	1.07 (1.01 to 1.13)	0.031
**BLD**				
Main model	0.98 (0.93 to 1.03)	0.365	1.01 (0.95 to 1.07)	0.714
+ PLT	1.00 (0.95 to 1.06)	0.996	1.02 (0.97 to 1.09)	0.402
+ MPV	0.98 (0.93 to 1.03)	0.430	1.01 (0.95 to 1.07)	0.707
**BLT**				
Main model	0.90 (0.85 to 0.95)	0.0002	0.97 (0.91 to 1.03)	0.273
+ PLT	0.92 (0.87 to 0.97)	0.004	0.98 (0.92 to 1.04)	0.527
+ MPV	0.90 (0.85 to 0.95)	0.0003	0.97 (0.91 to 1.03)	0.276

Axis: 0.8 1.0 1.2 (Men); 0.8 1.0 1.2 (Women)

Supplementary Figure S5 Associations of anthropometric indices and liver function tests with lung cancer risk: adjustment for platelet parameters

AFI – allometric fat-mass index; **ALI** – allometric lean-mass index; **ALP** – alkaline phosphatase; **ALT** – alanine aminotransferase; **AST** – aspartate aminotransferase; **BLD** – direct (conjugated) bilirubin; **BLT** – total bilirubin; **BMI** – body mass index; **CI** – confidence interval; **GGT** – gamma-glutamyl transferase; **HR** – hazard ratio; **MPV** – mean platelet volume; **PLT** – platelet count; **SD** – standard deviation**; cases** – number of lung cancer cases; **rate** – incidence rate per $1*10^6$ person years; **p-value** – Wald test for the individual term.

Main model – Cox proportional hazards models including each anthropometric index or liver function test individually as exposure (sex-specific z-scores, value minus mean divided by SD, after log-transformation for biomarkers), stratified by age at recruitment, region, and smoking status and intensity, and adjusted for height, recent weight gain, alcohol consumption, physical activity, Townsend deprivation index, family history of lung cancer, time of blood collection, fasting time, diabetes, and use of lipid-lowering drugs, antihypertensive drugs, antiaggregant/anticoagulants, and paracetamol, and in women, menopausal status, and hormone replacement therapy use.

+ PLT / + MPV – main model additionally adjusted either for PLT or for MPV (sex-specific z-scores after log-transformation).

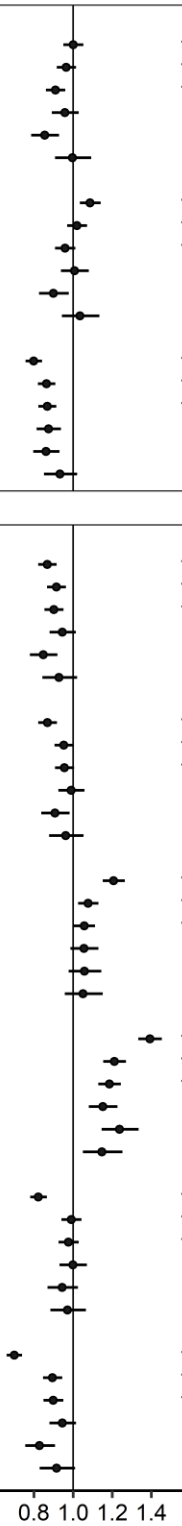

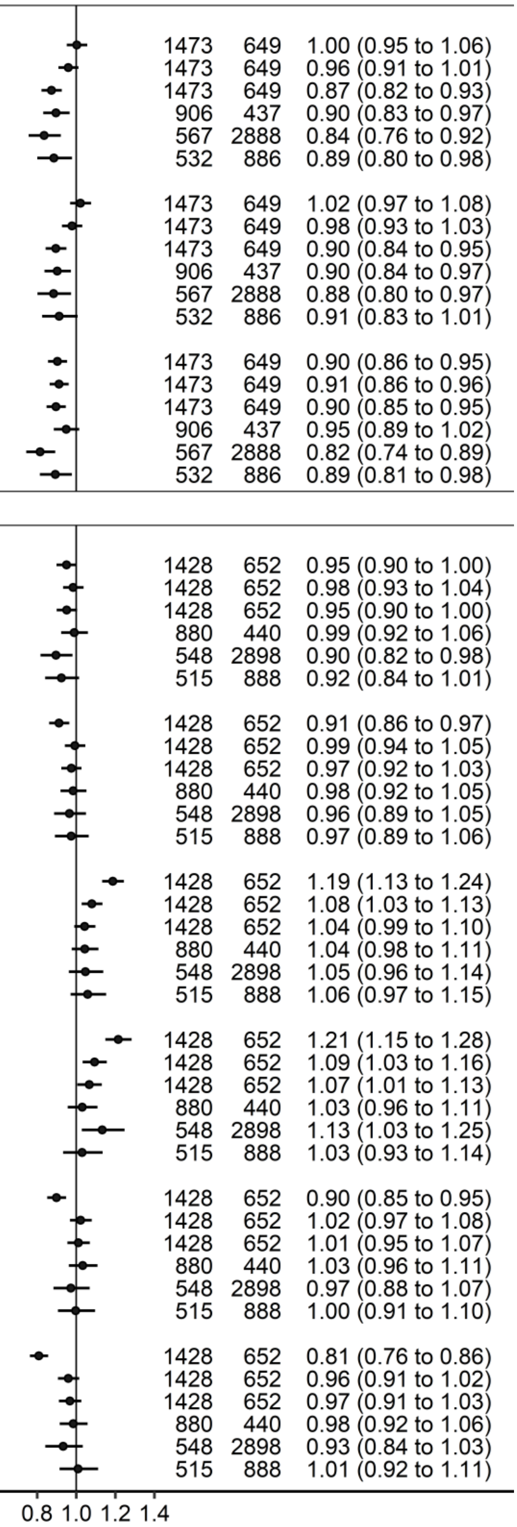

	Men cases	Men rate	Men HR (95% CI) (per one SD increase)	Women cases	Women rate	Women HR (95% CI) (per one SD increase)
**Anthropometric indices**						
**BMI**						
Age only	1573	810	1.00 (0.95 to 1.05)	1473	649	1.00 (0.95 to 1.06)
+ Smoking	1573	810	0.96 (0.92 to 1.01)	1473	649	0.96 (0.91 to 1.01)
Main model	1573	810	0.91 (0.86 to 0.96)	1473	649	0.87 (0.82 to 0.93)
Non-smokers	899	526	0.96 (0.89 to 1.03)	906	437	0.90 (0.83 to 0.97)
Current smokers	674	2916	0.85 (0.79 to 0.93)	567	2888	0.84 (0.76 to 0.92)
FUP≥8 years	528	1053	1.00 (0.91 to 1.09)	532	886	0.89 (0.80 to 0.98)
**AFI**						
Age only	1573	810	1.09 (1.03 to 1.14)	1473	649	1.02 (0.97 to 1.08)
+ Smoking	1573	810	1.02 (0.97 to 1.07)	1473	649	0.98 (0.93 to 1.03)
Main model	1573	810	0.96 (0.91 to 1.01)	1473	649	0.90 (0.84 to 0.95)
Non-smokers	899	526	1.01 (0.94 to 1.08)	906	437	0.90 (0.84 to 0.97)
Current smokers	674	2916	0.90 (0.83 to 0.98)	567	2888	0.88 (0.80 to 0.97)
FUP≥8 years	528	1053	1.03 (0.94 to 1.13)	532	886	0.91 (0.83 to 1.01)
**ALI**						
Age only	1573	810	0.80 (0.76 to 0.84)	1473	649	0.90 (0.86 to 0.95)
+ Smoking	1573	810	0.86 (0.82 to 0.91)	1473	649	0.91 (0.86 to 0.96)
Main model	1573	810	0.87 (0.82 to 0.91)	1473	649	0.90 (0.85 to 0.95)
Non-smokers	899	526	0.87 (0.81 to 0.94)	906	437	0.95 (0.89 to 1.02)
Current smokers	674	2916	0.86 (0.80 to 0.93)	567	2888	0.82 (0.74 to 0.89)
FUP≥8 years	528	1053	0.93 (0.85 to 1.02)	532	886	0.89 (0.81 to 0.98)
**Liver function tests**						
**ALT**						
Age only	1541	819	0.87 (0.82 to 0.92)	1428	652	0.95 (0.90 to 1.00)
+ Smoking	1541	819	0.91 (0.87 to 0.96)	1428	652	0.98 (0.93 to 1.04)
Main model	1541	819	0.90 (0.85 to 0.95)	1428	652	0.95 (0.90 to 1.00)
Non-smokers	885	534	0.94 (0.88 to 1.01)	880	440	0.99 (0.92 to 1.06)
Current smokers	656	2920	0.85 (0.78 to 0.92)	548	2898	0.90 (0.82 to 0.98)
FUP≥8 years	505	1037	0.93 (0.84 to 1.02)	515	888	0.92 (0.84 to 1.01)
**AST**						
Age only	1541	819	0.87 (0.82 to 0.92)	1428	652	0.91 (0.86 to 0.97)
+ Smoking	1541	819	0.95 (0.90 to 1.00)	1428	652	0.99 (0.94 to 1.05)
Main model	1541	819	0.95 (0.91 to 1.01)	1428	652	0.97 (0.92 to 1.03)
Non-smokers	885	534	0.99 (0.92 to 1.06)	880	440	0.98 (0.92 to 1.05)
Current smokers	656	2920	0.91 (0.84 to 0.98)	548	2898	0.96 (0.89 to 1.05)
FUP≥8 years	505	1037	0.96 (0.88 to 1.05)	515	888	0.97 (0.89 to 1.06)
**GGT**						
Age only	1541	819	1.21 (1.15 to 1.26)	1428	652	1.19 (1.13 to 1.24)
+ Smoking	1541	819	1.08 (1.03 to 1.13)	1428	652	1.08 (1.03 to 1.13)
Main model	1541	819	1.06 (1.00 to 1.11)	1428	652	1.04 (0.99 to 1.10)
Non-smokers	885	534	1.06 (0.99 to 1.13)	880	440	1.04 (0.98 to 1.11)
Current smokers	656	2920	1.06 (0.98 to 1.14)	548	2898	1.05 (0.96 to 1.14)
FUP≥8 years	505	1037	1.05 (0.96 to 1.15)	515	888	1.06 (0.97 to 1.15)
**ALP**						
Age only	1541	819	1.39 (1.33 to 1.45)	1428	652	1.21 (1.15 to 1.28)
+ Smoking	1541	819	1.21 (1.15 to 1.27)	1428	652	1.09 (1.03 to 1.16)
Main model	1541	819	1.19 (1.13 to 1.24)	1428	652	1.07 (1.01 to 1.13)
Non-smokers	885	534	1.15 (1.08 to 1.23)	880	440	1.03 (0.96 to 1.11)
Current smokers	656	2920	1.24 (1.15 to 1.34)	548	2898	1.13 (1.03 to 1.25)
FUP≥8 years	505	1037	1.15 (1.05 to 1.25)	515	888	1.03 (0.93 to 1.14)
**BLD**						
Age only	1541	819	0.82 (0.78 to 0.87)	1428	652	0.90 (0.85 to 0.95)
+ Smoking	1541	819	0.99 (0.94 to 1.04)	1428	652	1.02 (0.97 to 1.08)
Main model	1541	819	0.98 (0.93 to 1.03)	1428	652	1.01 (0.95 to 1.07)
Non-smokers	885	534	1.00 (0.93 to 1.07)	880	440	1.03 (0.96 to 1.11)
Current smokers	656	2920	0.94 (0.87 to 1.02)	548	2898	0.97 (0.88 to 1.07)
FUP≥8 years	505	1037	0.97 (0.88 to 1.07)	515	888	1.00 (0.91 to 1.10)
**BLT**						
Age only	1541	819	0.70 (0.66 to 0.74)	1428	652	0.81 (0.76 to 0.86)
+ Smoking	1541	819	0.89 (0.85 to 0.94)	1428	652	0.96 (0.91 to 1.02)
Main model	1541	819	0.90 (0.85 to 0.95)	1428	652	0.97 (0.91 to 1.03)
Non-smokers	885	534	0.94 (0.88 to 1.01)	880	440	0.98 (0.92 to 1.06)
Current smokers	656	2920	0.83 (0.76 to 0.91)	548	2898	0.93 (0.84 to 1.03)
FUP≥8 years	505	1037	0.92 (0.83 to 1.01)	515	888	1.01 (0.92 to 1.11)

Supplementary Figure S6 Associations of anthropometric indices and liver function tests with lung cancer risk: sensitivity analyses

AFI – allometric fat-mass index; **ALI** – allometric lean-mass index; **ALP** – alkaline phosphatase; **ALT** – alanine aminotransferase; **AST** – aspartate aminotransferase; **BLD** – direct (conjugated)

bilirubin; **BLT** – total bilirubin; **BMI** – body mass index; **CI** – confidence interval; **FUP** – follow-up time; **GGT** – gamma-glutamyl transferase; **HR** – hazard ratio; **MPV** – mean platelet volume; **PLT** – platelet count; **SD** – standard deviation; **cases** – number of lung cancer cases; **rate** – incidence rate per $1*10^6$ person years.

Age only – Cox proportional hazards model including each anthropometric index or liver function test individually as exposure (sex-specific z-scores, value minus mean divided by SD, after log-transformation for biomarkers), stratified by age at recruitment but omitting the adjustment for smoking status and covariates.

+ Smoking – Cox proportional hazards model stratified by age at recruitment and smoking status and intensity but omitting the adjustment for covariates.

Main model – multivariable Cox proportional hazards model, stratified by age at recruitment, region, and smoking status and intensity, and adjusted for height, recent weight gain, alcohol consumption, physical activity, Townsend deprivation index, family history of lung cancer, time of blood collection, fasting time, diabetes, and use of lipid-lowering drugs, antihypertensive drugs, antiaggregant/anticoagulants, and paracetamol, and in women, menopausal status, and hormone replacement therapy use.

Non-smokers / Current smokers – main model in groups according to smoking status, combining never and former smokers as non-smokers due to the limited number of lung cancer cases in never smokers.

FUP≥8 years – main model in participants with at least 8 years of follow-up and entry time lagged with 8 years.

	cases	rate	HR (95% CI)	p-value	cases	rate	HR (95% CI)	p-value
			**PLT−BMI**				**MPV−BMI**	
**Men**								
Low-Low	392	618	reference		561	853	reference	
High-Low	631	951	1.39 (1.22 to 1.58)	<0.0001	462	722	0.85 (0.75 to 0.96)	0.010
Low-High	294	913	1.11 (0.95 to 1.29)	0.203	238	768	0.76 (0.65 to 0.88)	0.0004
High-High	256	797	1.01 (0.86 to 1.18)	0.930	312	936	0.86 (0.74 to 0.99)	0.034
**Women**								
Low-Low	404	510	1.14 (0.99 to 1.31)	0.070	465	612	0.96 (0.85 to 1.09)	0.568
High-Low	527	726	1.41 (1.23 to 1.61)	<0.0001	466	614	0.99 (0.88 to 1.13)	0.918
Low-High	245	734	1.08 (0.91 to 1.27)	0.383	243	654	0.82 (0.70 to 0.96)	0.012
High-High	297	713	1.17 (1.00 to 1.36)	0.057	299	789	0.89 (0.77 to 1.04)	0.136
			**PLT−AFI**				**MPV−AFI**	
**Men**								
Low-Low	367	575	reference		516	783	reference	
High-Low	579	873	1.38 (1.21 to 1.57)	<0.0001	430	669	0.86 (0.75 to 0.97)	0.017
Low-High	319	1001	1.14 (0.98 to 1.32)	0.102	283	916	0.84 (0.72 to 0.97)	0.017
High-High	308	959	1.11 (0.95 to 1.30)	0.184	344	1040	0.90 (0.78 to 1.04)	0.139
**Women**								
Low-Low	404	510	1.16 (1.01 to 1.34)	0.039	459	604	0.99 (0.87 to 1.12)	0.819
High-Low	517	712	1.42 (1.24 to 1.63)	<0.0001	462	609	1.02 (0.90 to 1.16)	0.786
Low-High	245	734	1.08 (0.92 to 1.28)	0.343	249	671	0.85 (0.72 to 0.99)	0.036
High-High	307	737	1.20 (1.02 to 1.40)	0.025	303	799	0.92 (0.79 to 1.06)	0.254
			**PLT−ALT**				**MPV−ALT**	
**Men**								
Low-Low	455	742	reference		620	989	reference	
High-Low	698	1101	1.35 (1.20 to 1.52)	<0.0001	533	859	0.86 (0.77 to 0.97)	0.012
Low-High	206	656	1.00 (0.85 to 1.18)	0.998	175	564	0.73 (0.62 to 0.87)	0.0003
High-High	182	569	0.94 (0.79 to 1.12)	0.468	213	658	0.79 (0.67 to 0.92)	0.003
**Women**								
Low-Low	402	546	1.10 (0.96 to 1.26)	0.190	455	628	0.94 (0.83 to 1.07)	0.339
High-Low	528	727	1.33 (1.16 to 1.51)	<0.0001	475	644	0.97 (0.85 to 1.09)	0.587
Low-High	221	634	1.04 (0.89 to 1.23)	0.603	233	632	0.85 (0.73 to 0.99)	0.038
High-High	277	733	1.21 (1.04 to 1.41)	0.015	265	741	0.92 (0.80 to 1.07)	0.293

0.6 0.8 1.0 1.2 1.4 1.6   0.6 0.8 1.0 1.2

Supplementary Figure S7 Three-way cross-classifications according to sex, platelet parameters, and one of BMI, AFI, or ALT

AFI – allometric fat-mass index (cut-off men: ≥13.703; women: ≥15.119); **ALT** – alanine aminotransferase (cut-off men: ≥28.65; women: ≥20.61 IU/L); **BMI** – body mass index (cut-off men: ≥28.982; women: ≥28.144 kg/m^2); **CI** – confidence interval; **HR** – hazard ratio; **MPV** – mean platelet volume (cut-off men: ≥9.17; women: ≥9.25 fL); **PLT** – platelet count (cut-off men: ≥234.0; women: ≥261.4*10^9/L); **RERI** – relative excess risk from interaction (additive interaction); **cases** – number of lung cancer cases; **rate** – incidence rate per 1*10^6 person years; **p-value** – p-value for RERI derived with the delta method or p-value from Wald test for the individual term.

Cox proportional hazards models including jointly men and women with a three-way cross-classification between sex, one of PLT or MPV (dichotomised at the sex-specific median), and one of BMI, AFI, or ALT (dichotomised at the upper sex-specific tertile cut-off), stratified by age at recruitment, region, and smoking status and intensity, and adjusted for height, recent weight gain, alcohol consumption, physical activity, Townsend deprivation index, family history of lung cancer, time of blood collection, fasting time, diabetes, and use of lipid-lowering drugs, antihypertensive drugs, antiaggregant/anticoagulants, and paracetamol (note that the adjustment for menopausal status and hormone replacement therapy use was omitted because it was defined only in women). Category low-low-men was used as reference.

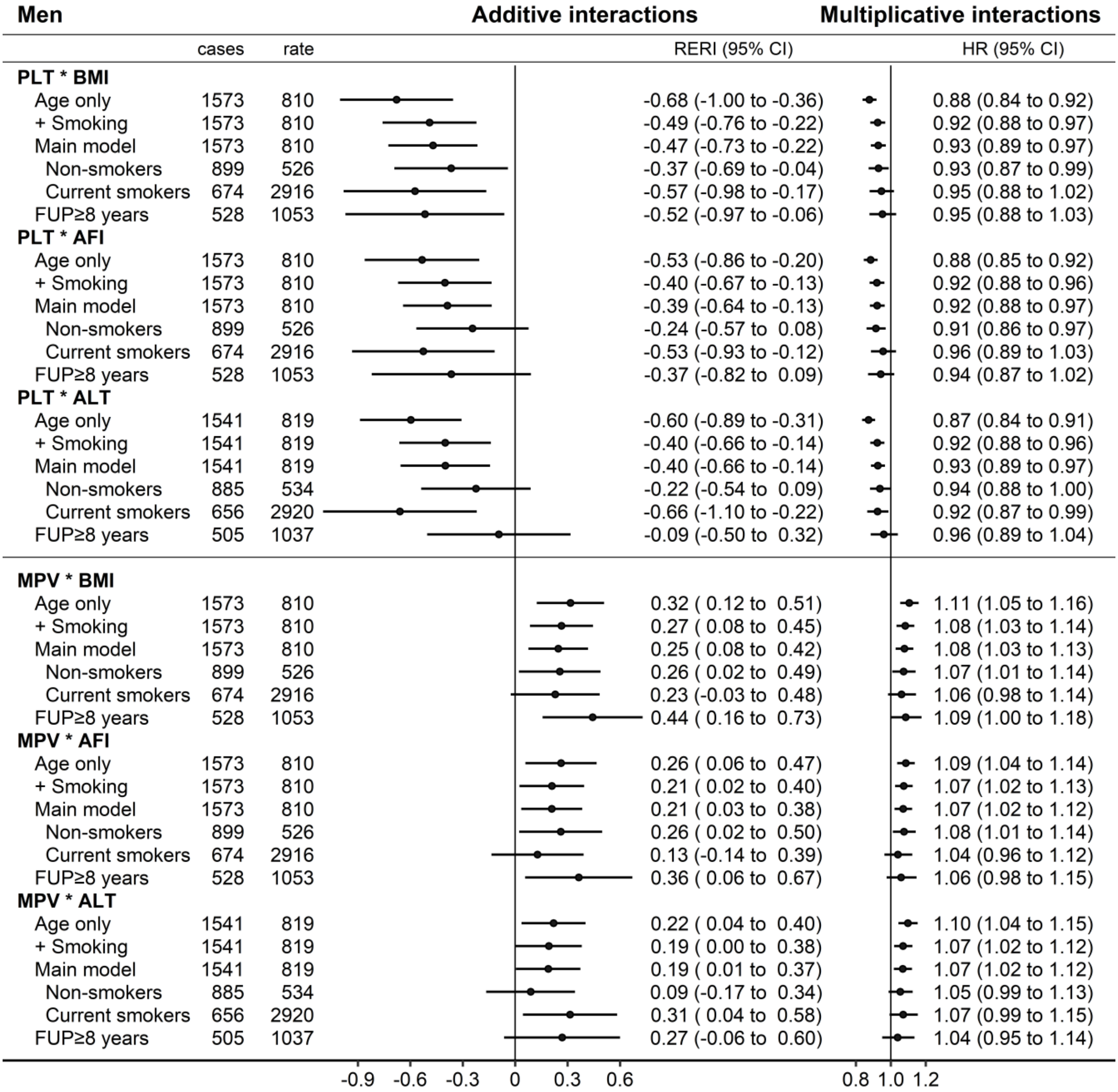

Supplementary Figure S8 Interactions with platelet parameters: sensitivity analyses (men)

AFI – allometric fat-mass index (cut-off: ≥13.703); **ALT** – alanine aminotransferase (cut-off: ≥28.65 IU/L); **BMI** – body mass index (cut-off: ≥28.982 kg/m^2); **CI** – confidence interval; **FUP** – follow-up time; **HR** – hazard ratio; **MPV** – mean platelet volume (cut-off: ≥9.17 fL); **PLT** – platelet count (cut-off: ≥234.0*10^9/L); **RERI** – relative excess risk from interaction; **SD** – standard deviation; **cases** – number of lung cancer cases; **rate** – incidence rate per 1*10^6 person years.

Additive interactions – RERI for two-way cross-classifications in men between one of PLT or MPV (dichotomised at the sex-specific median) and one of BMI, AFI, or ALT (dichotomised at the upper sex-specific tertile cut-off).

Multiplicative interactions – HR estimates in men for the interaction term between one of PLT or MPV and one of BMI, AFI, or ALT (sex-specific z-scores, value minus mean divided by SD, after log-transformation for biomarkers).

Age only – Cox proportional hazards model stratified only by age at recruitment and omitting the adjustment for smoking and covariates.

+ Smoking – Cox proportional hazards model stratified by age at recruitment and smoking status and intensity and omitting the adjustment for covariates.

Main model – multivariable Cox proportional hazards model, stratified by age at recruitment, region, and smoking status and intensity, and adjusted for height, recent weight gain, alcohol consumption, physical activity, Townsend deprivation index, family history of lung cancer, time of blood collection, fasting time, diabetes, and use of lipid-lowering drugs, antihypertensive drugs, antiaggregant/anticoagulants, and paracetamol.

Non-smokers / Current smokers – main model in groups according to smoking status, combining never and former smokers as non-smokers due to the limited number of lung cancer cases in never smokers.

FUP≥8 years – main model in participants with at least 8 years of follow-up and entry time lagged with 8 years.

References

References from the main document:

3. Christakoudi S, Tsilidis KK, Evangelou E, Riboli E. Interactions of platelets with obesity in relation to lung cancer risk in the UK Biobank cohort. *Respir Res*. 2023;24(1):249. https://doi.org/10.1186/s12931-023-02561-9

15. Christakoudi S, Tsilidis KK, Evangelou E, Riboli E. A Body Shape Index (ABSI), hip index, and risk of cancer in the UK Biobank cohort. *Cancer Med*. 2021;10(16):5614-28. https://doi.org/10.1002/cam4.4097

17. Sizoo D, de Heide LJM, Emous M, van Zutphen T, Navis G, van Beek AP. Measuring Muscle Mass and Strength in Obesity: a Review of Various Methods. *Obes Surg*. 2021;31(1):384-93. https://doi.org/10.1007/s11695-020-05082-2

18. Johnson Stoklossa CA, Forhan M, Padwal RS, Gonzalez MC, Prado CM. Practical Considerations for Body Composition Assessment of Adults with Class II/III Obesity Using Bioelectrical Impedance Analysis or Dual-Energy X-Ray Absorptiometry. *Curr Obes Rep*. 2016;5(4):389-96. https://doi.org/10.1007/s13679-016-0228-5

21. Wei R, Wang J, Su M, Jia E, Chen S, Chen T et al. Missing Value Imputation Approach for Mass Spectrometry-based Metabolomics Data. *Sci Rep*. 2018;8(1):663. https://doi.org/10.1038/s41598-017-19120-0

23. Lunn M, McNeil D. Applying Cox regression to competing risks. *Biometrics*. 1995;51(2):524-32.

39. Sylow L, Tokarz VL, Richter EA, Klip A. The many actions of insulin in skeletal muscle, the paramount tissue determining glycemia. *Cell Metab*. 2021;33(4):758-80. https://doi.org/10.1016/j.cmet.2021.03.020

40. Bouchard C, Tchernof A, Tremblay A. Predictors of body composition and body energy changes in response to chronic overfeeding. *Int J Obes (Lond)*. 2014;38(2):236-42. https://doi.org/10.1038/ijo.2013.77

Supplementary references:

53. Christakoudi S, Riboli E, Evangelou E, Tsilidis KK. Associations of body shape index (ABSI) and hip index with liver, metabolic, and inflammatory biomarkers in the UK Biobank cohort. *Sci Rep*. 2022;12(1):8812. https://doi.org/10.1038/s41598-022-12284-4

54. Christakoudi S, Riboli E, Evangelou E, Tsilidis KK. Associations of body shape phenotypes with sex steroids and their binding proteins in the UK Biobank cohort. *Sci Rep*. 2022;12(1):10774. https://doi.org/10.1038/s41598-022-14439-9
